# Personalized B cell response to the *Lactobacillus rhamnosus GG* probiotic in healthy human subjects: a randomized trial

**DOI:** 10.1080/19490976.2020.1854639

**Published:** 2020-12-04

**Authors:** Jette Bornholdt, Christa Broholm, Yun Chen, Alfredo Rago, Stine Sloth, Jakob Hendel, Cathrine Melsæther, Christina V. Müller, Maria Juul Nielsen, Jesper Strickertsson, Lars Engelholm, Kristoffer Vitting-Seerup, Kim B. Jensen, Adam Baker, Albin Sandelin

**Affiliations:** a Biotech Research and Innovation Centre, University of Copenhagen, Copenhagen N, Denmark; b The Bioinformatics Centre, Department of Biology, University of Copenhagen, Copenhagen N, Denmark; c Human Health Discovery, Hørsholm, Denmark; d Gastro Unit, Herlev Hospital, University of Copenhagen, Herlev, Denmark; e Finsen Laboratory, University of Copenhagen, Copenhagen N, Denmark; f Danish Cancer Society, Copenhagen Ø, Denmark; g Novo Nordisk Foundation Center for Stem Cell Biology, DanStem, University of Copenhagen, Copenhagen N, Denmark

**Keywords:** *Lactobacillus rhamnosus* GG, immediate in vivo effect, probiotics, human transcriptomics, B cell activation

## Abstract

The specific effects of administering live probiotics in the human gut are not well characterized. To this end, we investigated the immediate effect of *Lactobacillus rhamnosus* GG (LGG) in the jejunum of 27 healthy volunteers 2 h after ingestion using a combination of global RNA sequencing of human biopsies and bacterial DNA sequencing in a multi-visit, randomized, cross-over design (ClinicalTrials.gov number NCT03140878). While LGG was detectable in jejunum after 2 h in treated subjects, the gene expression response vs. placebo was subtle if assessed across all subjects. However, clustering analysis revealed that one-third of subjects exhibited a strong and consistent LGG response involving hundreds of genes, where genes related to B cell activation were upregulated, consistent with prior results in mice. Immunohistochemistry and single cell-based deconvolution analyses showed that this B cell signature likely is due to activation and proliferation of existing B cells rather than B cell immigration to the tissue. Our results indicate that the LGG strain has an immediate effect in the human gut in a subpopulation of individuals. In extension, our data strongly suggest that studies on *in vivo* probiotic effects in humans require large cohorts and must take individual variation into account.

## Introduction

Dietary live bacteria, collectively called probiotics, are one of the most commonly consumed dietary supplements^1^ and are recommended to patients by up to 60% of health-care providers.^2^ The rationale for taking probiotics includes protection against infections, e.g., respiratory tract infections^3^ and alleviation of antibiotic-associated diarrhea.^4^ However, there is a growing need to understand the mode of action of specific probiotics and their direct impact on human physiology.

Almost 2000 clinical studies have been conducted to examine the health benefits of probiotics.^5,6^ Whereas meta-analyses support clinical benefits of the consumption of probiotics in specific populations which are at risk of developing diseases, e.g., patients at risk of developing clostridium difficile^6^ associated diarrhea, it has not been possible to make generalized conclusions on health benefits in a variety of conditions and conflicting results have been reported. Clinical trials examining probiotics are complicated by the substantial and largely unexplored inter-individual human variability, which beyond genetics may be explained by factors such as age, diet, antibiotics usage, use of food supplements, underlying disease^7^ and by baseline microbiome patterns.^8^ A rigorous examination of specific and molecular probiotic responses and personalized effects on the intestinal mucosa in larger human cohorts would be of great value for researchers and health-care professionals to understand the mode of action and dynamics of probiotics in human health science.

*Lactobacillus rhamnosus* GG (LGG) bears the most substantial and scientific support for its clinical efficacy. The clinical effects seen with LGG intervention are primarily within immunity and include decreased incidence of respiratory infections,^9,10^ enhanced antibody formation during viral infection^11^ and improved antibody response to vaccines.^12^ *In vitro* studies suggest that LGG may exert those effects through diverse modes of actions of which some are ascribed to its immune-regulatory effects. LGG is recognized by the *TLR2* receptor^13^ and thereby is able to induce a cascade of immunological events in the epithelial cells and/or antigen-presenting cells. LGG has been shown to activate the transcription factor NF-kB, which is one of the central activators of innate immune response, and the Toll-like receptors *TLR1* and *TLR2*, which mediate bacterial recognition and cellular signaling.^14^

Despite the already extensive scientific literature covering diverse aspects of LGG, studies examining gene expression activated *in vivo* at the intestinal mucosa after LGG consumption in humans are sparse. Pagnini et al.^15^ showed that LGG ingestion for 7 days resulted in a dose-dependent decrease of *TNF* (TNAɑ) and *IL7* genes in ulcerative colitis patients, as measured by qPCR in colon biopsies. Van Baarlen and coworkers^16^ used microarrays to measure gene expression in endoscopies sampled from duodenal biopsies of 7 subjects 6 h after consumption of three different probiotics, including LGG. The gene expression change following LGG consumption was highly varied between individuals, and related to this, the overall gene expression response was small (no genes were significantly differentially expressed after multiple testing correction). This suggests that either the response in all individuals is small or that sub-groups of individuals respond differently. To address this important question, well-designed experiments with substantially larger cohorts are necessary.

To this end, we hypothesized that LGG would elicit gene expression responses in jejunal tissue shortly after consumption, and sought to understand what these responses were, and whether such responses would be generic or only present in a subgroup of subjects. We used a randomized placebo-controlled cross-over design study to investigate the 2-h jejunal mucosal response to ingestion of 450bn LGG in 27 human volunteers. We correlated our findings to anthropometric measures and microbiome composition in luminal fluid obtained from jejunum to dissect personalized responses. Our results indicate that LGG reached the jejunum in 2 h, and around 1/3 of individuals had a clear response to LGG stimulation when compared to placebo, with a gene expression signature consistent with B cell activation, which in turn is consistent with previous studies in mouse models.

## Results

### Overview of experimental design

**Figure 1.**
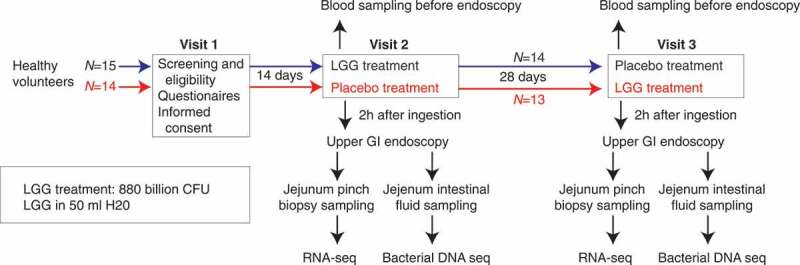
Experimental design. A total of 29 healthy volunteers were enrolled in a cross-over design. At visit 1, volunteers were assessed for eligibility, given a questionnaire and informed consent was acquired. At visit 2, 15 subjects (blue) ingested LGG solution and remaining 15 subjects ingested placebo solution (red). Two hours after ingestion an upper GI endoscopy was performed: biopsies and intestinal fluid from jejunum were sampled for RNA-seq and bacterial DNA sequencing, respectively. At visit 3, the same procedure as in visit 2 was repeated, but subjects given LGG in visit two received placebo and vice versa (see color highlights). Two subjects developed conditions that required medication between visits 2 and 3 and were therefore excluded from the study, so the final number of subjects was 27 as indicated

**Figure 2.**
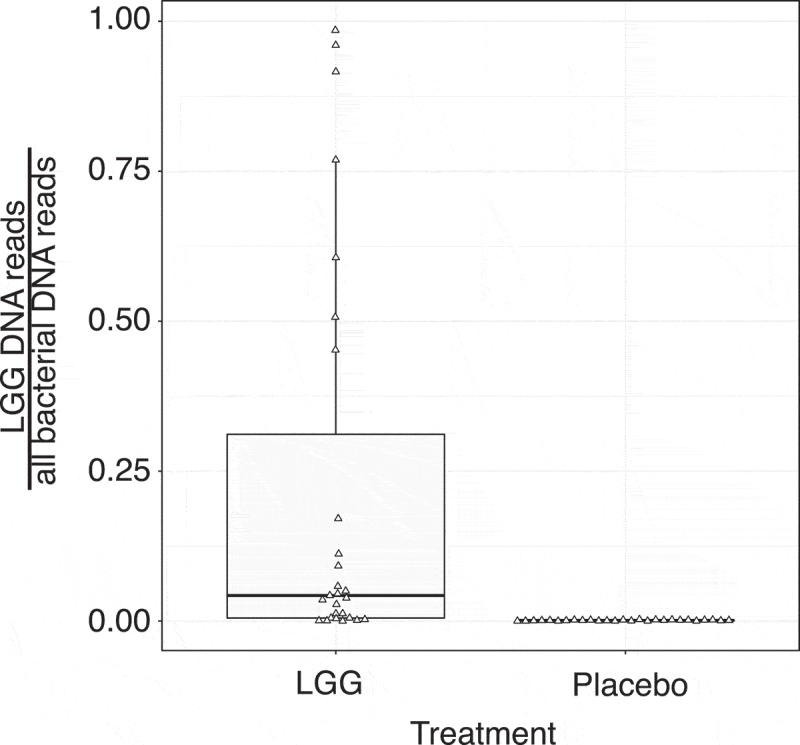
Fraction of LGG reads from bacterial DNA sequencing. Y-axis shows the fraction of reads originating from LGG vs. all bacteria in luminal fluid from jejunum taken 2 h after LGG or placebo digestion as boxplots. Triangles show individual samples (*N* = 23; two runs failed). X-axis shows treatment

For a clinical trial, we recruited 29 volunteer individuals who participated in three visits (Table 1). In visit 1, screening took place and informed consent was obtained (Figure 1). In visit 2, half of the individuals ingested LGG, while the other half ingested placebo solution, and jejunum biopsies and luminal fluids were taken 2 h later for both groups. The same procedure was repeated at visit 3, 28 days later, where individuals that received LGG in visit 2 received placebo, and vice versa (Figure 1), constituting a randomized cross-over experimental design. RNA was extracted from biopsies and subjected to gene expression analysis by global poly-A-selected RNA sequencing (RNA-seq) (Supplementary Table 1). The bacterial composition of the luminal fluid was determined by global bacterial DNA sequencing. Mapping these DNA reads to the LGG genome showed that LGG could only be detected in small intestine luminal fluid from LGG-treated individuals (Figure 2), strongly indicating that LGG reached the jejunum 2 h after ingestion, with no cross-contamination in non-LGG treated subjects.

**Table 1.** Clinical characteristics of the final 27 volunteers recruited. Average values and standard deviations are shown

Clinical characteristics	Mean ± SD
Age (years)	24.4 ± 4
Sex (male/female)	14/13
Height (cm)	173.3 ± 11
Weight (kg)	69.6 ± 14
BMI (Kg/m^2^)	22.9 ± 3
Systolic blood pressure	128.3 ± 10
Diastolic blood pressure	81.6 ± 8
Resting pulse (BPM)	73.7 ± 15
Alcohol intake (units/week)	3.2 ± 4
Time spent on sport (h/week)	5.3 ± 4
*CRP (mg/L)	1.2 ± 2
HbA1c (mmol/mol)	31.6 ± 3

*CRP was only analyzed at the first intervention day (visit 2, see main text).

### Clustering analysis revealed a LGG-responding sub-group

**Figure 3.**
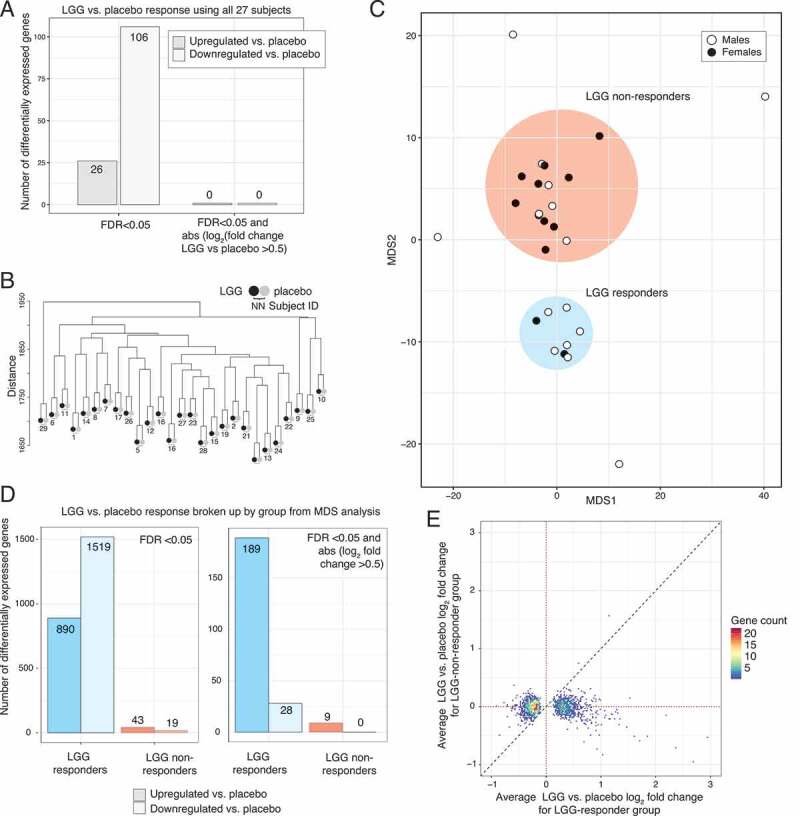
Differential expression analysis of LGG vs. placebo response. (a) Expression analysis using all subjects as a single group. Y-axis shows the number of differentially expressed genes (LGG vs. placebo), as a function of cutoffs based only on significance, or significance and effect size (X-axis). Opaque colors show up-regulation, pale colors show down-regulation. Numbers on bars show the number of up/down-regulated genes. (b) Hierarchical clustering of normalized RNA-seq libraries. Y-axis shows Euclidean distance in the tree, created by complete linkage. Each subject is represented by two leaves, colored by treatment. Note that libraries always cluster by subject, not treatment (indicated by subject number below leaf pairs). (c) Multidimensional scaling (MDS) plot based on log_2_ LGG/control RNA-seq expression values. Axes represent dimensions 1 and 2. Dots represent subjects, colored by sex. Two major groups (defined as LGG-responders and non-LGG-responders, based on subsequent analysis, see main text and panel D) were observed, as indicated by color. Three subjects were outside these groups and were considered as outliers (excluded in subsequent analyses). The subset of genes (*N* = 1389) that showed LGG responsiveness were used for MDS analysis (see Methods). (d) Expression analysis using groups defined in panel C. Y-axis shows the number of differentially expressed genes (LGG vs. placebo) within groups defined in panel C (X-axis; blue bars show results for LGG-responders, red bars show results for non-LGG-responders, where solid color indicate up-regulated genes and and pale colors down-regulated genes). Upper panel shows the results of analysis using *FDR*<0.05 as cutoff, lower panel shows the results of analysis using *FDR*<0.05 and absolute log_2_ fold change >0.5. Numbers on bars show the number of up/down-regulated genes. (e) Relation between LGG vs. placebo expression change in LGG-responders and non-LGG-responders. Y-axis shows the average RNA-seq log_2_ fold change (LGG vs. placebo) in non-LGG-responder subjects. X-axis shows the average RNA-seq log_2_ fold change (LGG vs. placebo) in LGG-responder subjects. Only genes that were differentially expressed in LGG-responders are shown (*FDR*<0.05). Dots are colored by the number of genes falling into respective plot area. Dotted lines show log_2_ fold change of 0 (no change) on both axes. Dashed line shows the diagonal (Y = X)

We used normalized gene expression estimates from the RNA-seq data analyzed by DeSeq2^17^ to compare the effect of LGG vs. placebo ingestion, initially treating all subjects as one group. We found 106 and 25 up- and down-regulated genes (DeSeq2 *FDR* <0.05), respectively. Importantly, these changes in gene expression, while statistically significant, had low effect sizes (LGG vs. placebo log_2_ fold change): if requiring an absolute log_2_ fold change >0.5 (corresponding to an up/down-regulation of a factor of approximately >1.4), no differentially expressed genes were detected (Figure 3a). Thus, the average gene expression response to LGG across the subject population was not substantial.

Differential expression analysis is highly influenced by variance within groups. Given the results above, we reasoned that individual variation may mask sub-groups of individuals that may respond differently to LGG. To compare the contribution of LGG treatment and difference between individuals, we hierarchically clustered RNA-seq data from all subjects resulting from LGG and placebo treatments. Samples clustered strongly according to individual rather than treatment, suggesting that even in a relatively homogenous subject group such as this, individual differences are larger than the treatment effects (Figure 3b). To explore whether sub-groups of individuals with similar LGG response patterns existed, we performed a multidimensional scaling (MDS) analysis of the paired RNA-seq data sets (specifically, the log_2_ fold change between LGG and placebo for every subject and LGG-responding genes). This revealed two distinct groups of 8 (group A) and 15 subjects (group B), and 4 outlier subjects (Figure 3c). The grouping was not significantly associated with whether subjects were given LGG on visit 2 or 3 (*P* > .05, Fisher’s exact test).

Differential expression analysis performed as above but within each group separately showed that group B had few differentially expressed genes in response to LGG (43 up-regulated, 19 down-regulated at *FDR*<0.05, and only 9 up-regulated genes when also requiring an absolute log_2_ fold change >0.5). Conversely, group A showed a much larger response: 890 upregulated and 1519 downregulated genes (*FDR*<0.05), and 189 upregulated and 28 downregulated genes when also requiring an absolute log_2_ fold change >0.5 (Figure 3d). Importantly, up- or down-regulated genes in group A did not overall show an expression change in a consistent direction in group B, and the most highly upregulated genes (log_2_ fold change >1 LGG vs. placebo) in group A were in all but one case less expressed in LGG than placebo in group B (log_2_ fold change <0) (Figure 3e). Thus, group B subjects could not be characterized as having a similar but weaker response compared to group A. Because of these results, we will refer to group A as ‘LGG-responders’ and group B as ‘non-LGG-responders’.

### Characterization of LGG-responding genes

**Figure 4.**
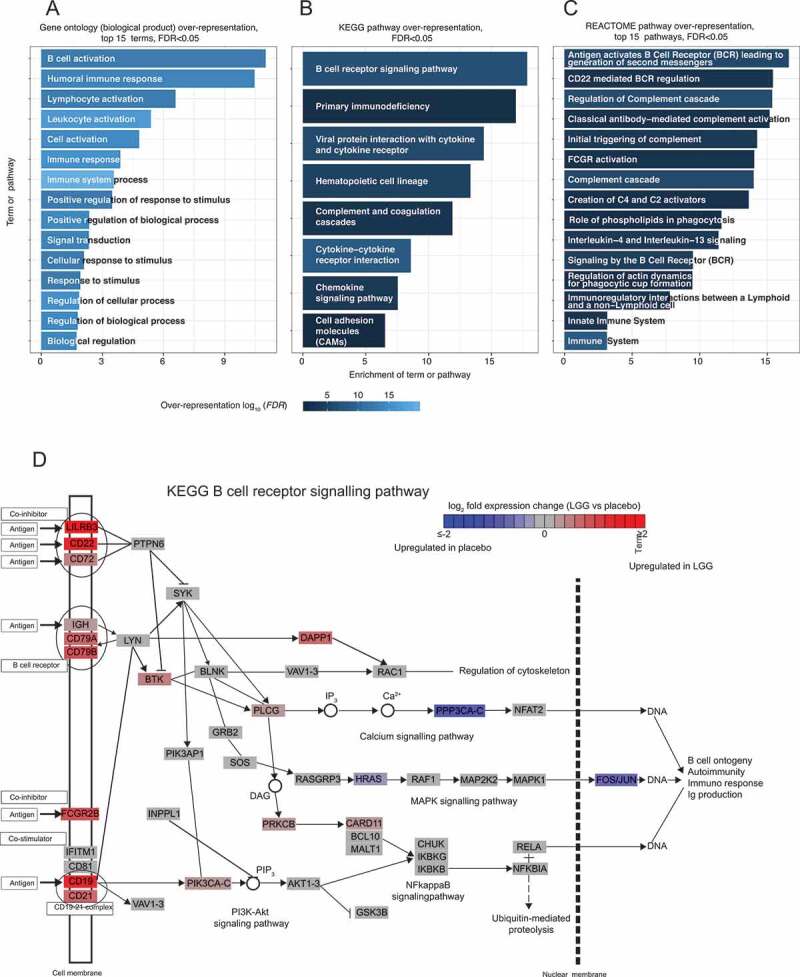
Gene ontology (GO) and pathway analysis of genes upregulated after LGG treatment in the LGG-responder group. (a) Over-representation of GO terms. Y-axis shows top 10 Biological Process GO terms (*FDR*<0.05 and enrichment score >3), sorted after enrichment score (X-axis). Bars are colored by over-representation *FDR* on -log_10_ scale. (b) Over-representation of KEGG pathways. Plot is organized as in A, but shows over-represented KEGG pathways (*FDR*<0.05 and enrichment score >3). (c) Over-representation of REACTOME pathways. Plot is organized as in A, but shows top 15 over-represented REACTOME pathways (*FDR*<0.05 and enrichment score >3). (d) LGG response of gene in the B cell response pathway. Pathway schematic is based on the KEGG^14^ database. Boxes indicate genes or complexes, colored by their LGG response (average log_2_ fold change LGG vs placebo); note that colors are capped at log_2_ fold change +-2

To focus on the most biologically relevant changes in the LGG-responder group, we analyzed the more strict gene set defined above (*FDR* <0.05 and log_2_ fold change >0.5; see Supplementary Tables 2–3). The genes upregulated after LGG treatment were strongly enriched for Gene Ontology (GO) terms related to B cell activation, immune system, immune response, and leukocyte activation (Figure 4a). In agreement with the above, KEGG^18^ and REACTOME^19^ pathway over-representation analysis showed a strong over-representation of pathways related to B cell activation through the B cell receptor (first or second most-enriched term in respective analysis, Figure 4b–c), where the expression of membrane receptors of the pathway was elevated upon LGG treatment, including *CD22, CD19, CD21, CD79a(IGa) CD79B(IGb), FCGR2B* genes which included the B cell receptor components (Figure 4d).

**Figure 5.**
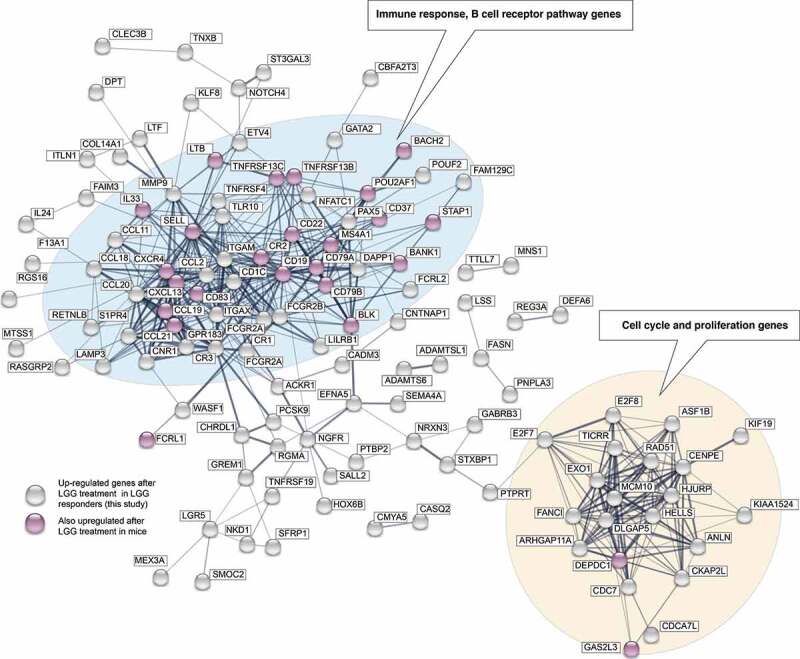
STRING interaction analysis plot of LGG-upregulated genes. Circles indicate genes upregulated after LGG treatment in the LGG -responsive group (*FDR*<0.05, log_2_ fold change >0.5; gene names within boxes). Lines between genes represent evidence of interaction or co-expression (from the STRING database), where thicker lines indicate stronger evidence. Gene circle color indicates whether the genes were also upregulated following LGG treatment in mouse.^21^ Genes with no connections in the STRING database are not shown. Two major interaction clusters are evident, dominated by immune response genes (including B cell response), and proliferation/cell cycle genes (blue and beige background, respectively)

STRING interaction analysis^20^ showed that these genes formed two substantial interaction clusters, one dominated by DNA replication, repair and cell cycle-related genes including *MCM2, MCM4, CDC45, CNPE* and another dominated by immune response genes, including B cell activation-associated genes (Figure 5). Notably, there was no over-representation of inflammation or defense response GO terms or pathways, although some chemokines such as *CXCL13, CCL18* and *CCL19* were upregulated (discussed further below). Because B cell activation through the B cell receptor and coreceptors CD19/CD21 leads to cell cycle entry and proliferation,^22^ the second gene cluster in the STRING analysis, dominated by cell-cycle-related genes, may also reflect the B cell response. LGG-downregulated genes were enriched for GO terms and pathways associated with vitamin D, fatty acid and retinoid metabolism (Supplementary Figure 1A-C).

Suzuki et al.^21^ previously showed that ingestion of LGG in mouse models also resulted in activation of B cell response genes in biopsies taken from the terminal ileum, where higher LGG doses led to higher numbers of upregulated B cell response genes. Even though the experimental design was different from ours (LGG was administered daily over 2 weeks), there was a statistically significant overlap of 26 up-regulated genes between our study and the mouse study using the highest LGG concentration (*P* = 8.046e-14, two-sided Fisher’s exact test, Supplementary Figure 1D). The shared set of 26 up-regulated genes was involved in B cell activation (genes highlighted in pink in Figure 5). Altogether, these results show a consistent response between LGG-responding humans and mice in terms of LGG response, involving B cell activation genes.

RNA expression differences in tissue may either be due to changes in expression in existing cells, or reflecting changes in cellular composition in the tissue. Thus, the B cell expression signature we observed may be derived from existing B cells reacting to LGG, or an influx of B cells as a response to LGG exposure. Immunohistochemical detection of B cells based on CD20 on randomly sampled jejunal biopsies showed that both LGG-responders and non-LGG-responders displayed B-cell clusters (Supplementary Figure 2). To rule out that the cell composition between responder and non-LGG-responder groups before or after LGG treatment drives the observed differences, we estimated the proportions of each cell type present in the samples by cell deconvolution based on the RNA-seq data. Briefly, cell deconvolution estimates the relative abundance of cell types in each sample by quantifying the expression of cell-specific gene markers (see Supplementary Figure 3, Supplementary Table 4 and Methods). Importantly, we found no evidence supporting differences in cell composition between LGG-responder and non-LGG-responder subjects (39 tests, 2-sample Wilcoxon Rank Sum Test, all *FDR* > 0.05). Similarly, we found no significant change in the frequency of any cell types between placebo- and LGG-treated subjects (39 tests, paired Mann–Whitney 2-sided tests, all *FDR* > 0.05).

Thus, histology and cell convolution analyses both showed that the responses we observed in our experiments were unlikely to be due to initial differences in cell composition between individuals or to changes in tissue cell composition after LGG exposure. A more likely explanation is that we observed early LGG responses of B cells already present in the gut tissue, which would also fit the time frame of our analysis (we measure RNA expression in jejunum 2 h after digestion, which means that the effective exposure time between LGG and gut cells measured was likely substantially less). Due to this short time frame, most of the RNA-seq observations may reflect changes on RNA level that had not yet manifested on protein level.

The specific up-regulated genes also make it likely that we observed a B cell activation rather than influx. In particular, two of the five most upregulated genes are involved in the differentiation of B cells, including *MS4A1* (encoding the CD20 protein)^23^ and *PAX5,^24^* and as discussed above, the large induction of cell cycle-related genes observed (Figure 5a) is characteristic of B-cell proliferation rather than influx. It is interesting to note that the B cell attractants *CXCL13, CCL19, CCL18* were also among the most upregulated genes despite no evidence for B cell influx; we hypothesize the upregulation may herald immune cell influx occurring at a later state.

### Analysis of subject and metagenomics data of LGG-responders vs. non-LGG-responders

An important question is why a given individual is an LGG-responder or non-LGG-responder. We noted that males were distributed equally between LGG-responders (6/11) and non-LGG-responders (5/11), while only 2/13 females were LGG-responders, but the time of LGG treatment (visit 2 or 3) had no impact when taking sex distributions into account. We investigated other collected parameters, including age, systolic and diastolic blood pressure, resting pulse, weight, height, self-reported average alcohol consumption and physical exercise level. Using a logistic regression model, none of these parameters could alone, or in combination, accurately predict whether a given individual was an LGG-responder or not (*P* > .05 in all cases, logistic regression), although one should note that logistic regression with few samples has low power.

As discussed above, because we sampled intestinal fluid in parallel, we could also sequence bacterial DNA for 23 subjects (LGG and placebo-treated). We found no significant difference in the fraction of DNA reads from LGG in LGG-treated LGG-responders and LGG-treated non-LGG-responders (*P* = 1, two-sided Mann–Whitney U test), indicating that the speed of LGG transfer is similar between LGG-responders and non-LGG-responders.

Moreover, when removing LGG reads from the analysis, we found no statistically significant difference in bacterial diversity or relative bacterial abundance between the responders and non-LGG-responders (before or after treatment) regardless of level analyzed (phylum, class, order, family, genus and species; all two-sided Mann–Whitney *U* tests *P* > .05). Thus, it is presently unclear what determines the LGG responsiveness: genetic differences between individuals or unmeasured physiological parameters may be partially responsible. Moreover, it remains a possibility that all individuals respond to LGG but some respond slower and are therefore not detected within the 2 h time frame of the experiment (see Discussion).

## Discussion

In the present study, we have investigated the short-term *in vivo* gene expression response of LGG ingestion in 27 healthy individuals in the jejunum part of the small intestine. We observed a substantial gene expression response in roughly one-third of the analyzed subjects. To our knowledge, this is the first study to robustly show an early *in vivo* gene expression response and immediate action of probiotics in general, and in particular for LGG in humans.

Aside from a sex bias (only 15% of females were LGG-responders, compared to 55% of males), we could not conclusively identify subject parameters (e.g. blood pressure, BMI, etc.) that could explain the difference in response. Similarly, there were no obvious differences in bacterial overall composition before or after treatment between LGG-responder and non-LGG-responder groups, and no substantial difference in LGG abundance after treatment in the jejunum between LGG-responder and non-LGG-responder groups. Response differences may be associated with parameters not measured, differences in life history, diet, or genetics between individuals (also see below for further discussion in the light of comparisons with animal models). In order to pinpoint genetic variants associated with LGG response (quantitative trait loci analysis), even larger human cohorts are necessary.

One of the strongest expression signatures in LGG-responders was an upregulation of B cell activation-associated genes. Immunohistochemistry and cell deconvolution analysis did not show evidence of B cell influx as a response to LGG, or changes in cell composition between LGG-responders and non-LGG-responders, but rather supported a model where the observed transcription response is a reflection of existing B cells sensing and reacting to LGG. This is consistent with the small time frame of the experiment and does not preclude cell composition changes at later time points or at prolonged exposures.

To our knowledge, LGG or any probiotic response in this time frame has never been previously shown in humans, although the observed LGG-induced B cell activation signature is consistent with results from mouse models.^21^ A core set of B cell activation-associated genes upregulated in our study was also upregulated in mouse jejunum biopsies following LGG treatment, and the number of shared upregulated genes was a function of LGG concentration in mice. It is thus highly unlikely that the B cell activation response in LGG-responders is a spurious effect. It is interesting to note that as opposed to our results in humans, in mice no obvious subgroups (e.g. LGG-responders or non-LGG-responders) were reported.^21^ This may be due to the high genetic, and life history, similarities between the mice analyzed compared to humans. Alternatively, it may be due to the difference in experimental designs. In our study, LGG was ingested only once and the response measured after 2 h, in mice, LGG was ingested daily over 15 days. Thus, our results in humans likely reflect acute LGG response rather than continuous response as in mice, which would also fit with the induction of cell cycle genes in the human but not mouse samples (Figure 3a), due to B cell activation and subsequent early cell division. As we saw no substantial difference in LGG abundance in jejunum 2 h after exposure in responders *vs*. non-LGG-responders, it is unlikely that the speed of LGG transfer can explain our results, but it is possible that non-LGG-responders may have delayed LGG response. This reflects a limitation in our study: only the 2 h time point was assessed, and only in jejunum samples using a single, relatively high LGG dose. Given our result, an interesting follow-up would be to either sample continuously after ingestion (which would be technically difficult) or to use a design where LGG is ingested continuously over longer time periods, similar to the mouse experiment or as in ref.^15^ but using a paired design and global RNA expression profiling.

As a summary, our study has demonstrated clear gene expression response of gut cells in the jejunum 2 h after ingestion of the probiotic LGG – a response dominated by B cell activation which only manifested in one-third of the tested cohort. We cannot at present ascertain whether the LGG-induced B cell activation response is beneficial, but it is interesting to note that studies have shown that administration of LGG augmented B cell response, which in turn had beneficial effects in immunization to rotaviruses in pig models.^25,26^ Because we could not establish the underlying reason for the differential response between groups, it is at present difficult to assess the clinical impact of our observation, or how the observation can directly lead to improved probiotics strains. Therefore, beyond establishing that a subset of healthy individuals have a distinct B cell response to LGG as early as 2 h in jejunum which indicates that LGG exerts immunomodulatory effects on very short time scales, the most important impact of our study for future work may be our demonstration that improved experimental designs of clinical trials of probiotic products is necessary to reach meaningful conclusions. The identification of LGG-responders/non-LGG-responder groups in our data would not have been possible with smaller cohort sizes, exemplified by the lack of robust LGG responses on gene expression level using seven subjects in a previous study,^16^ and also required a paired design where the same individual is assessed before and after treatment. Thus, because of the large individual human variation in probiotic response, much larger cohort sizes than employed previously are necessary, in combination with careful, paired experimental designs. Such improved designs and the notion that sub-groups of responders may exist and that such groups have to be stratified in analysis, is necessary for assessing the clinical effect probiotics and the development of improved probiotic strains.

## Methods

### Human subjects and ethics

Twenty-nine healthy individuals (males and females) between the ages of 18 to 35, with a BMI <30 kg/m^2^ (Table 1) were included in the study. The number of subjects was based on a previous study analyzing gene expression data from gut tissue.^27^ Individuals were excluded if they were pregnant or breastfeeding, had diagnoses or disorders requiring chronic or intermittent medicine, had taken systemic antibiotics or steroids or antimicrobial medication in the last 4 months or suffered from intolerances. Included subjects were not allowed to consume fermented dairy products 2 weeks prior to visit 2 and during the study period. Subjects were instructed to abstain from all types of medicine, probiotics, vitamins, minerals and other food supplements and to maintain normal lifestyle and dietary habits. We encouraged subjects not to travel in the study period and abstain from alcohol 2 days prior to visit 2 and 3. One subject deviated from the dietary instructions at visit 3, but it was decided to include the subject in the analyses regardless. Two subjects developed conditions that required medication between visit 2 and 3 and were excluded from the study. Thus, the final data set consisted of paired samples from 27 individuals. In visit 1, questionnaires and consent forms were completed and inclusion/exclusion criteria were checked. Participants were given oral and written information about experimental procedures in visit 2 and 3 before giving their written informed consent. No severe adverse events were observed during the study. The study was approved by the regional ethical committee (H-17002470) and performed according to the Declaration of Helsinki. All data analyses were performed blinded. All authors had access to the study data and reviewed and approved the final manuscript. The trial was registered at ClincialTrials.gov with the identifier NCT03140878. CONSORT checklist, flow diagram and study protocol are available in Supplementary Text 1.

### Randomization and blinding

The randomization was generated by a person not involved in the study using RANCODE version 3.6 (IDV) and SAS version 8.2 (SAS Institute Inc.). Group randomization was performed in a 1:1 ratio in blocks of six. Study products were labeled according to the randomization lists and only identified by the randomization number. Subject allocation was performed by the investigator in consecutive order by assigning eligible subjects the first available randomization number. All the subjects, investigators, site staff and sponsor staff involved in the study were blinded until the final database was locked. Only the study supply coordinator at Chr. Hansen A/S had access to the randomization list to perform labeling of the study products.

### Experimental design overview

On visit 2 and 3, individuals arrived at the hospital after having fasted since midnight. In a paired cross-over design (Figure 1), in visit 2, 15 randomly selected subjects ingested 450 Bn LGG (see below) dissolved in 50 ml water, while the remaining 14 subjects ingested 50 ml placebo solution. Subjects were left resting for 2 hours before sedation with nurse administered propofol sedation (NAPS) and upper GI endoscopy was performed using a pediatric colonoscope (PCF-190, Olympus). Intestinal fluid (1–5 ml) was aspirated from jejunum using a short rinse of saline solution for bacterial DNA sequencing. Hereafter mucosal pinch biopsies (approximately 15 mg each) were obtained, using endoscopic forceps. Biopsies were snap-frozen on dry ice and stored at −80 C for RNA sequencing. In the third visit, 28 days later, the procedure in visit 2 was repeated but the subjects that ingested LGG received placebo, and vice versa.

### Test product

The investigational product, freeze-dried *Lactobacillus rhamnosus* GG produced at Chr. Hansen A/S, commercially known as (LGG®), and the placebo product, was dissolved in 50 ml water immediately before ingestion by the volunteers. Cell counts of the product consumed by each volunteer were calculated at 885 Billion Colony Forming Units (CFU) LGG®. There was a small difference in the materials used as cryoprotectant/filler in the placebo and LGG powder; the placebo contained maltodextrin (filler, 130 mg) and microcrystalline cellulose (filler, 45 mg), whereas the LGG contained sucrose (cryoprotectant, 16 mg), maltodextrin (cryoprotectant, 10 mg), microcrystalline cellulose (filler, 178 mg) and LGG (active, 54 mg). However, there was no evidence of a sugar response in placebo vs. LGG treatment: only 0.9% (13/1466) and 0.5% (3/649) of previously identified glucose-responding genes in HepG2 and Caco2,^28,29^ respectively, were significantly changing in the individuals receiving LGG vs. placebo (LGG-responder sub-group, Supplementary Figure 4)

### Blood sampling

Blood samples were obtained from the antecubital vein during visit 2 and 3, before ingestion of LGG or placebo for CRP and Hba1c analysis.

### Sequencing read pre-processing

Trim Galore (https://github.com/FelixKrueger/TrimGalore, version 0.4.4) was used to trim low-quality 3ʹ read ends (Phred score lower than 25) and the first 5 bp of 5ʹ ends with biased nucleotide composition.

### Bacterial DNA sequencing and metagenomics analyses

One ml luminal fluid sample was immediately spun down at 1000 G for 5 min at 4°C. Supernatant was removed and the pellets were frozen and shipped to BaseClear, NL, where DNA was extracted and sequenced. The average sequencing depth of the libraries was 6,255,219 (S.D ± 12,117,819). Trimmed metagenomics reads (see above) were mapped to the LGG reference genome (ASM2650v1) to measure the fraction of LGG reads. Non-human-mappable reads (hg19) were used as input to MetaPhlAn2.^30^ Microbiota compositions were quantified using default parameters at six different taxonomy ranks (phylum, class, order, family, genus, species). To filter out low-quality libraries and taxonomies, samples with no taxonomy detected and taxonomies that were covered by <3 samples were removed. Relative taxonomy abundance per sample was used for checking composition differences between LGG-responders and non-LGG-responders. Analyses covered all the six taxonomy ranks, and included i) Taxonomy changes before and after LGG treatment, split by the responder group and the non-LGG-responder group. ii) Differences of taxonomy abundance between responders and non-LGG-responders, including average abundance of placebo condition and LGG condition, only placebo condition and only LGG condition. iii) Alpha diversity (diversity() function from the R vegan package) differences between the responder group and the non-LGG-responder group. iv) Beta diversity differences between responders and non-LGG-responders. For each taxonomy rank, the abundance matrix was transformed to a presence/absence matrix. The binary matrix was then fed to base.pair() function from the R betapart package^30,31^ to calculate Jaccard pairwise dissimilarity between subjects (beta diversity). For statistical tests, two-sided Mann–Whitney tests were used in analysis i)–iii) (paired tests in i), and the betadisper() function from the vegan package in combination with ANOVA tests were used in iv).

### RNA purification, sequencing and analysis

RNA were extracted using PureLink® RNA Mini Kit (Ambion^TM^, Life Technologies) and 1% 2-mercaptoethanol (Sigma). Biopsies were homogenized in 700 µl lysis buffer using Ultra-Turrax® (IKA Works, Inc) and purified according to manufacturer’s instructions with on-column DNase treatment (AmbionTM, Life Technologies) and 40 µl elution volume. Samples were shipped to Novogene (Hong Kong) Co., LTD for polyA-selected stranded 150 bp paired-end RNA sequencing (RNA-seq). All samples passed the service providers quality criteria for quantity and purity. The average sequencing depth of the libraries was 46,798,632 (S.D ± 6,310,076). Trimmed RNA-seq reads were quantified to gene expression using salmon^32^ v.0.8.2 with parameters “–gcBias – seqBias” and the GRCh38 assembly. The expression matrix is supplied as Supplementary Table 1. An exploratory analysis of possible batch effects was performed (Supplementary Text 1). We used DESeq2 to call differentially expressed genes between control and LGG-treated samples (~ subject + condition). Hierarchical clustering was done on the whole gene expression set excluding lowly expressed outliers whose sum of expression across all libraries was <-10 on log_2_ scale. In Figure 3c, variance stabilizing transformation was applied on salmon-quantified gene expression counts. Log_2_ fold change of LGG versus control for each gene and each subject was calculated in order to investigate individual LGG responses. One thousand three hundred and eighty-nine genes with absolute log_2_ LGG/control ≥1.4 were used in the MDS analysis to identify LGG-responders and non-LGG-responders. Clustering and MDS were done by using hclust() and cmdscale() functions in R.

### Gene ontology and KEGG overrepresentation analysis and visualization

GO, KEGG and REACTOME pathway over-representation analysis made using the gprofiler2 R package^33^ with standard settings, using all detected genes in the RNA-seq data as background. Enrichment was defined as (intersect/gene set of interest)/(total GO term or pathway size/size of background gene set). KEGG pathway visualization was made using the pathview R package.^34^

### Comparison between human and mouse expression data

We selected significantly upregulated genes from the supplemental data from ref.^21^ using the same cutoff criteria as in our conservative RNA-seq analysis (*FDR* <0.05, log_2_ fold change >0.5). We used expression data from mouse terminal ileum with the highest LGG concentration (10e9 LGG). We used the HomoloGene database^35^ through the homologene R package (https://CRAN.R-project.org/package=homologene) to identify orthologous human-mouse gene pairs.

### Immunohistochemistry

Biopsies were fixed in 4% paraformaldehyde and stored at 4°C. At minimum 7 days prior to embedding in Tissue Tek (Sakura Finetek) biopsies were placed in 70% ethanol. 3.5 µm thick sections were deparaffinized in 2*10 min Tissue clear and dehydration. Serial sections of the biopsies were analyzed to ensure a thorough overview of B cell distribution throughout the collected tissue specimen. Sections were stained by Mayers Hematoxylene/Erosin (Histolab) according to the manufacturer’s protocol. Sections stained with CD20 antibody (M07755, clone L26, DAKO) was pre-treated with 98°C TEG-buffer (10 mM Tris, 0.5 mM EGTA, pH 9.0) for 15 min and 1% hydrogen peroxide (Merck) for 15 min. Sections were incubated with CD20 antibody (1:500) overnight at 4°C. Visualization was performed with Envision Mouse (K4001, DAKO) and NovaRED (VECTOR) according to manufacturer’s protocol. Sections were mounted using Tissue Tek tissue mount (Sakura Finetek).

### Cell-type deconvolution

We used dTangle^36^ to estimate changes in the cell-type composition present in each sample. dTangle works by assuming that increased expression of cell-type-specific marker genes is proportional to the increase of those cells in the sample. The analysis requires a set of markers for cell types of interest, and the slope of the linear relationship between gene expression and cell abundance, estimated from single cell expression data for each cell. We used single-cell RNA-seq data from colon biopsies of individuals with and without ulcerative colitis,^37^ after normalizing expression values for batch, individual and cell-cycle effects using limma.^38^ To increase robustness, we grouped together several sub-types of cells present in the original dataset, defining markers for 39 cell types (Supplementary Table 4). We assessed changes in cell proportion between groups (LGG-responders and non-LGG-responders) by two-sided Mann–Whitney tests. For assessing changes in cell proportions between LGG and placebo treatments, we used Mann–Whitney tests, paired by subject. We FDR-corrected all *P*-values.

## Supplementary Material

Supplemental Material

## References

[1] Clarke TC, Black LI, Stussman BJ, Barnes PM, Nahin RL. Trends in the use of complementary health approaches among adults: United States, 2002–2012. Natl Health Stat Report. 2015;79:1–16. PMC4573565 25671660

[2] Draper K, Ley C, Parsonnet J. Probiotic guidelines and physician practice: a cross-sectional survey and overview of the literature. Benef Microbes. 2017;8:507–519. doi:10.3920/BM2016.0146. 28618862

[3] Hao Q, Dong BR, Wu T. Probiotics for preventing acute upper respiratory tract infections. Cochrane Database Syst Rev. 2015;2:CD006895. 25927096 10.1002/14651858.CD006895.pub3

[4] Guo Q, Goldenberg JZ, Humphrey C, El Dib R, Johnston BC. Probiotics for the prevention of pediatric antibiotic-associated diarrhea. Cochrane Database Syst Rev. 2019;4:CD004827. 31039287 10.1002/14651858.CD004827.pub5 PMC6490796

[5] Kleerebezem M, Binda S, Bron PA, Gross G, Hill C, van Hylckama Vlieg JE, Lebeer S, Satokari R, Ouwehand AC. Understanding mode of action can drive the translational pipeline towards more reliable health benefits for probiotics. Curr Opin Biotechnol. 2019;56:55–60. doi:10.1016/j.copbio.2018.09.007. 30296737

[6] Shen NT, Maw A, Tmanova LL, Pino A, Ancy K, Crawford CV, Simon MS, Evans AT. Timely use of probiotics in hospitalized adults prevents clostridium difficile infection: a systematic review with meta-regression analysis. Gastroenterology. 2017;152:1889–900.e9. doi:10.1053/j.gastro.2017.02.003. 28192108

[7] Zmora N, Zeevi D, Korem T, Segal E, Elinav E. Taking it personally: personalized utilization of the human microbiome in health and disease. Cell Host Microbe. 2016;19:12–20. doi:10.1016/j.chom.2015.12.016. 26764593

[8] Zmora N, Zilberman-Schapira G, Suez J, Mor U, Dori-Bachash M, Bashiardes S, Kotler E, Zur M, Regev-Lehavi D, Brik RB-Z, et al. Personalized gut mucosal colonization resistance to empiric probiotics is associated with unique host and microbiome features. Cell. 2018;174:1388–405.e21. doi:10.1016/j.cell.2018.08.041. 30193112

[9] Hojsak I, Abdović S, Szajewska H, Milosević M, Krznarić Z, Kolacek S. Lactobacillus GG in the prevention of nosocomial gastrointestinal and respiratory tract infections. Pediatrics. 2010;125:e1171–7. doi:10.1542/peds.2009-2568. 20403940

[10] Hojsak I, Snovak N, Abdović S, Szajewska H, Misak Z, Kolacek S. Lactobacillus GG in the prevention of gastrointestinal and respiratory tract infections in children who attend day care centers: a randomized, double-blind, placebo-controlled trial. Clin Nutr. 2010;29:312–316. doi:10.1016/j.clnu.2009.09.008. 19896252

[11] Capurso L. Thirty years of Lactobacillus rhamnosus GG: a review. J Clin Gastroenterol. 2019;53(Suppl 1):S1–41. doi:10.1097/MCG.0000000000001170. 30741841

[12] Davidson LE, Fiorino A-M, Snydman DR, Hibberd PL. Lactobacillus GG as an immune adjuvant for live-attenuated influenza vaccine in healthy adults: a randomized double-blind placebo-controlled trial. Eur J Clin Nutr. 2011;65:501–507. doi:10.1038/ejcn.2010.289. 21285968 PMC3071884

[13] Miettinen M, Veckman V, Latvala S, Sareneva T, Matikainen S, Julkunen I. Live Lactobacillus rhamnosus and Streptococcus pyogenes differentially regulate Toll-like receptor (TLR) gene expression in human primary macrophages. J Leukoc Biol. 2008;84:1092–1100. doi:10.1189/jlb.1206737. 18625909

[14] Miettinen M, Lehtonen A, Julkunen I, Matikainen S. Lactobacilli and Streptococci activate NF-kappa B and STAT signaling pathways in human macrophages. J Immunol. 2000;164:3733–3740. doi:10.4049/jimmunol.164.7.3733. 10725732

[15] Pagnini C, Corleto VD, Martorelli M, Lanini C, D’Ambra G, Di Giulio E, Delle Fave G. Mucosal adhesion and anti-inflammatory effects of GG in the human colonic mucosa: a proof-of-concept study. World J Gastroenterol. 2018;24:4652–4662. doi:10.3748/wjg.v24.i41.4652. 30416313 PMC6224475

[16] van Baarlen P, Troost F, van der Meer C, Hooiveld G, Boekschoten M, Brummer RJM, Kleerebezem M. Human mucosal in vivo transcriptome responses to three lactobacilli indicate how probiotics may modulate human cellular pathways. Proc Natl Acad Sci U S A. 2011;108(Suppl 1):4562–4569. doi:10.1073/pnas.1000079107. 20823239 PMC3063594

[17] Love MI, Huber W, Anders S. Moderated estimation of fold change and dispersion for RNA-seq data with DESeq2. Genome Biol. 2014;15:550. doi:10.1186/s13059-014-0550-8. 25516281 PMC4302049

[18] Kanehisa M, Sato Y, Furumichi M, Morishima K, Tanabe M. New approach for understanding genome variations in KEGG. Nucleic Acids Res. 2019;47:D590–5. doi:10.1093/nar/gky962. 30321428 PMC6324070

[19] Jassal B, Matthews L, Viteri G, Gong C, Lorente P, Fabregat A, Sidiropoulos K, Cook J, Gillespie M, Haw R, et al. The reactome pathway knowledgebase. Nucleic Acids Res. 2020;48:D498–503. 31691815 10.1093/nar/gkz1031 PMC7145712

[20] Szklarczyk D, Gable AL, Lyon D, Junge A, Wyder S, Huerta-Cepas J, Simonovic M, Doncheva NT, Morris JH, Bork P, et al. STRING v11: protein-protein association networks with increased coverage, supporting functional discovery in genome-wide experimental datasets. Nucleic Acids Res. 2019;47:D607–13. doi:10.1093/nar/gky1131. 30476243 PMC6323986

[21] Suzuki C, Aoki-Yoshida A, Aoki R, Sasaki K, Takayama Y, Mizumachi K. The distinct effects of orally administered Lactobacillus rhamnosus GG and Lactococcus lactis subsp. lactis C59 on gene expression in the murine small intestine. PLoS One. 2017;12:e0188985. doi:10.1371/journal.pone.0188985. 29220366 PMC5722381

[22] Richards S, Watanabe C, Santos L, Craxton A, Clark EA. Regulation of B-cell entry into the cell cycle. Immunol Rev. 2008;224:183–200. doi:10.1111/j.1600-065X.2008.00652.x. 18759927 PMC2728081

[23] Cragg MS, Walshe CA, Ivanov AO, Glennie MJ. The biology of CD20 and its potential as a target for mAb therapy. Curr Dir Autoimmun. 2005;8:140–174. 15564720 10.1159/000082102

[24] Robichaud GA, Nardini M, Laflamme M, Cuperlovic-Culf M, Ouellette RJ. Human Pax-5 C-terminal isoforms possess distinct transactivation properties and are differentially modulated in normal and malignant B cells. J Biol Chem. 2004;279:49956–49963. doi:10.1074/jbc.M407171200. 15385562

[25] Kandasamy S, Chattha KS, Vlasova AN, Rajashekara G, Saif LJ. Lactobacilli and Bifidobacteria enhance mucosal B cell responses and differentially modulate systemic antibody responses to an oral human rotavirus vaccine in a neonatal gnotobiotic pig disease model. Gut Microbes. 2014;5:639–651. doi:10.4161/19490976.2014.969972. 25483333 PMC4615723

[26] Vlasova AN, Chattha KS, Kandasamy S, Liu Z, Esseili M, Shao L, Rajashekara G, Saif LJ. Lactobacilli and bifidobacteria promote immune homeostasis by modulating innate immune responses to human rotavirus in neonatal gnotobiotic pigs. PLoS One. 2013;8:e76962. doi:10.1371/journal.pone.0076962. 24098572 PMC3788735

[27] Boyd M, Thodberg M, Vitezic M, Bornholdt J, Vitting-Seerup K, Chen Y, Coskun M, Li Y, Lo BZS, Klausen P, et al. Characterization of the enhancer and promoter landscape of inflammatory bowel disease from human colon biopsies. Nat Commun. 2018;9:1661. doi:10.1038/s41467-018-03766-z. 29695774 PMC5916929

[28] Boztepe T, Gulec S. Investigation of the influence of high glucose on molecular and genetic responses: an study using a human intestine model. Genes Nutr. 2018;13:11. doi:10.1186/s12263-018-0602-x. 29736189 PMC5928582

[29] Jeong Y-S, Kim D, Lee YS, Kim H-J, Han J-Y, Im -S-S, Chong HK, Kwon J-K, Cho Y-H, Kim WK, et al. Integrated expression profiling and genome-wide analysis of ChREBP targets reveals the dual role for ChREBP in glucose-regulated gene expression. PLoS One. 2011;6:e22544. doi:10.1371/journal.pone.0022544. 21811631 PMC3141076

[30] Truong DT, Franzosa EA, Tickle TL, Scholz M, Weingart G, Pasolli E, Tett A, Huttenhower C, Segata N. MetaPhlAn2 for enhanced metagenomic taxonomic profiling. Nat Methods. 2015;12:902–903. doi:10.1038/nmeth.3589. 26418763

[31] Baselga A, Orme CDL. betapart: an R package for the study of beta diversity: betapart package. Methods Ecol Evol. 2012;3:808–812. doi:10.1111/j.2041-210X.2012.00224.x.

[32] Patro R, Duggal G, Love MI, Irizarry RA, Kingsford C. Salmon provides fast and bias-aware quantification of transcript expression. Nat Methods. 2017;14:417–419. doi:10.1038/nmeth.4197. 28263959 PMC5600148

[33] Reimand J, Arak T, Adler P, Kolberg L, Reisberg S, Peterson H, Vilo J. g: Profiler-a web server for functional interpretation of gene lists (2016 update). Nucleic Acids Res. 2016;44:W83–9. doi:10.1093/nar/gkw199. 27098042 PMC4987867

[34] Luo W, Brouwer C. Pathview: an R/Bioconductor package for pathway-based data integration and visualization. Bioinformatics. 2013;29:1830–1831. doi:10.1093/bioinformatics/btt285. 23740750 PMC3702256

[35] NCBI Resource Coordinators. Database resources of the National Center for Biotechnology Information. Nucleic Acids Res. 2016;44:D7–19. doi:10.1093/nar/gkv1290. 26615191 PMC4702911

[36] Hunt GJ, Freytag S, Bahlo M, Gagnon-Bartsch JA. dtangle: accurate and robust cell type deconvolution. Bioinformatics. 2019;35:2093–2099. doi:10.1093/bioinformatics/bty926. 30407492

[37] Smillie CS, Biton M, Ordovas-Montanes J, Sullivan KM, Burgin G, Graham DB, Herbst RH, Rogel N, Slyper M, Waldman J, et al. Intra- and inter-cellular rewiring of the human colon during ulcerative colitis. Cell. 2019;178:714–30.e22. doi:10.1016/j.cell.2019.06.029. 31348891 PMC6662628

[38] Ritchie ME, Phipson B, Wu D, Hu Y, Law CW, Shi W, Smyth GK. limma powers differential expression analyses for RNA-sequencing and microarray studies. Nucleic Acids Res. 2015;43:e47. doi:10.1093/nar/gkv007. 25605792 PMC4402510

