# Supplementary material for: Personalized B cell response to the Lactobacillus rhamnosus GG probiotic in healthy human subjects: a randomized trial
Source: Gut Microbes. 2020 Dec 4;12(1):1854639. doi: 10.1080/19490976.2020.1854639 (PMC7722709; doi:10.1080/19490976.2020.1854639)
Supplement: Supplemental Material [file KGMI_A_1854639_SM3886.zip › Supplementary information/All supplementary figures.pdf]

Figure S1

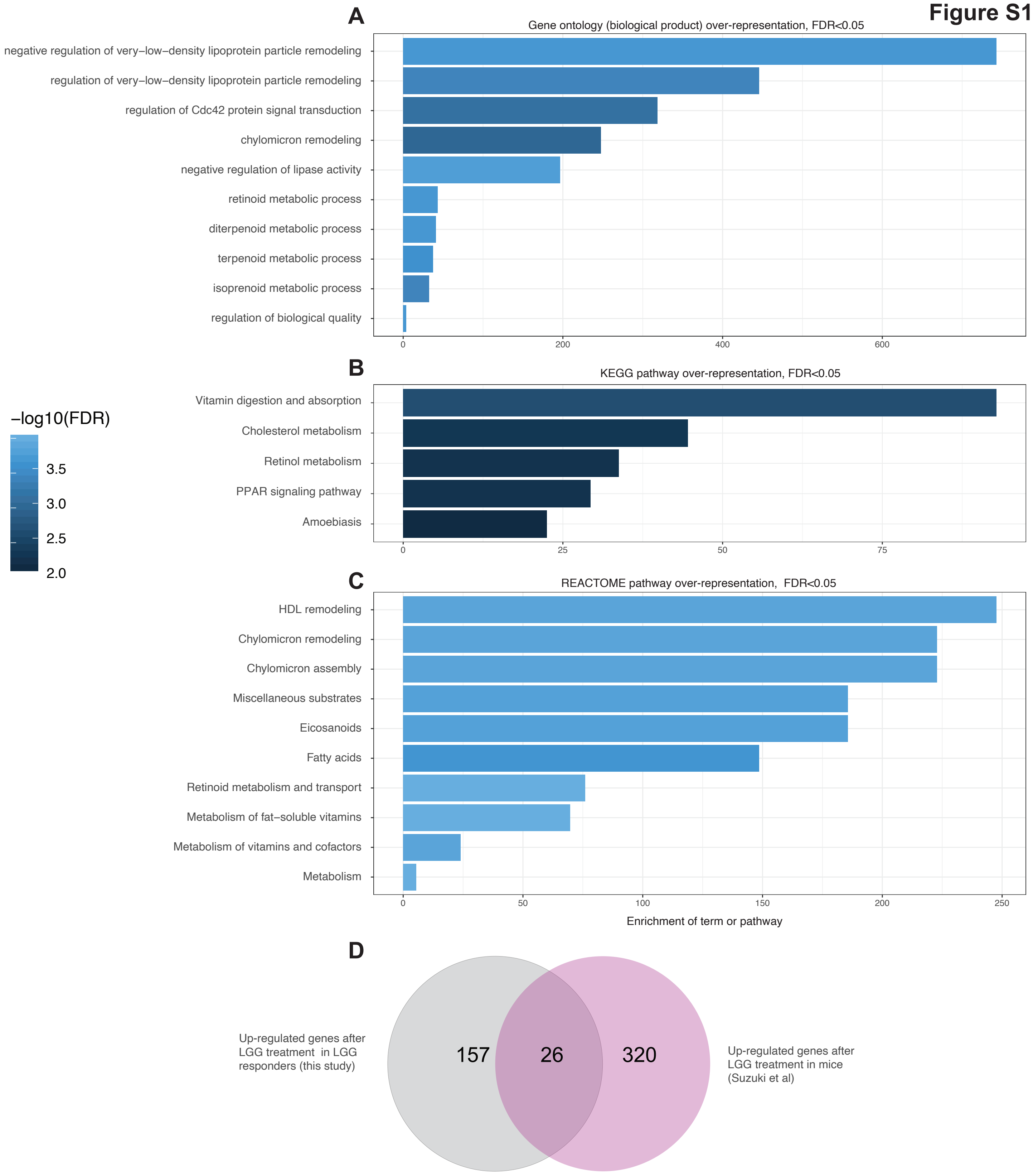

Gene ontology (GO) and pathway analysis of genes downregulated after LGG treatment in the LGG-responder group, and overlap with mouse studies

A: Over-representation of GO terms. Y axis shows top 10 Biological Process GO terms (FDR<0.05 and enrichment score >3), sorted after enrichment score (X axis). Bars are colored by over-representation FDR on -log10 scale.

B: Over-representation of KEGG pathways. Plot is organized as in A, but shows over-represented KEGG pathways (FDR<0.05 and enrichment score >3).

C: Over-representation of KEGG pathways. Plot is organized as in A, but shows top 15 over-represented REACTOME pathways (FDR<0.05 and enrichment score >3).

D: B: Overlap between human and mouse LGG response. Overlap between LGG-upregulated genes from the LGG-responsive group in this study and LGG-upregulated genes from a similar experiment in mice. Mouse genes are from Suzuki et al using the same differential expression cutoffs as in human and the 109 LGG dose. The 26 overlapping genes are highlighted in pink in Figure 5A.

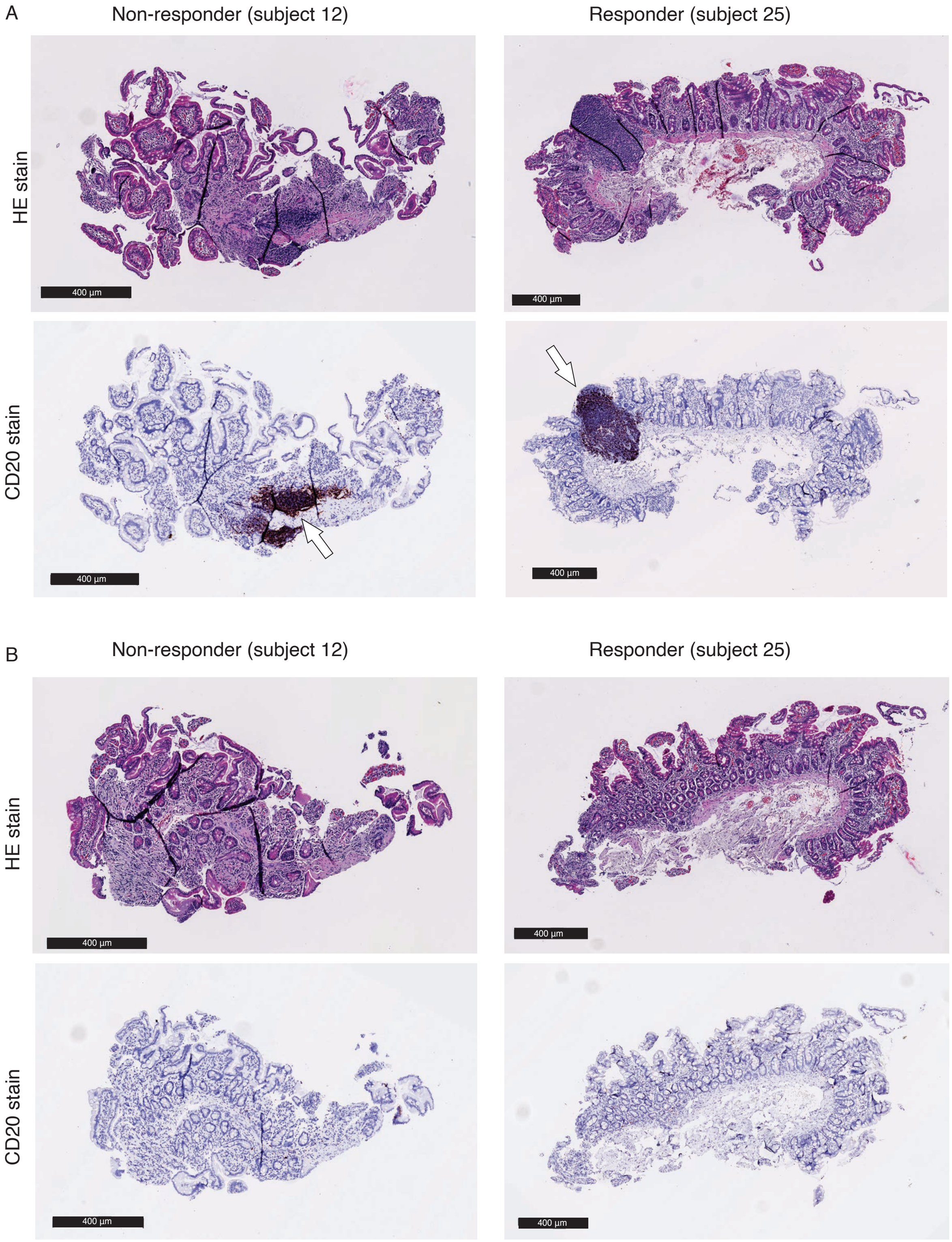

Immunohistochemical stainings for CD20 after LGG exposure.
HE and CD20 (B cell marker) stains from two randomly sampled jejunum biopsies from each subject group (LGG responders and non-responders) after LGG exposure are shown. The two top panels shows two adjacent sections (stained with HE and CD20, respectively) where a cluster of B-cells can be observed (indicated by arrows). The lower two panels show the same samples, but taken 10 sections away (105 um) to illustrate the heterogeneity of the biopsy, as the b-cell clusters are no longer visible.

Figure S3

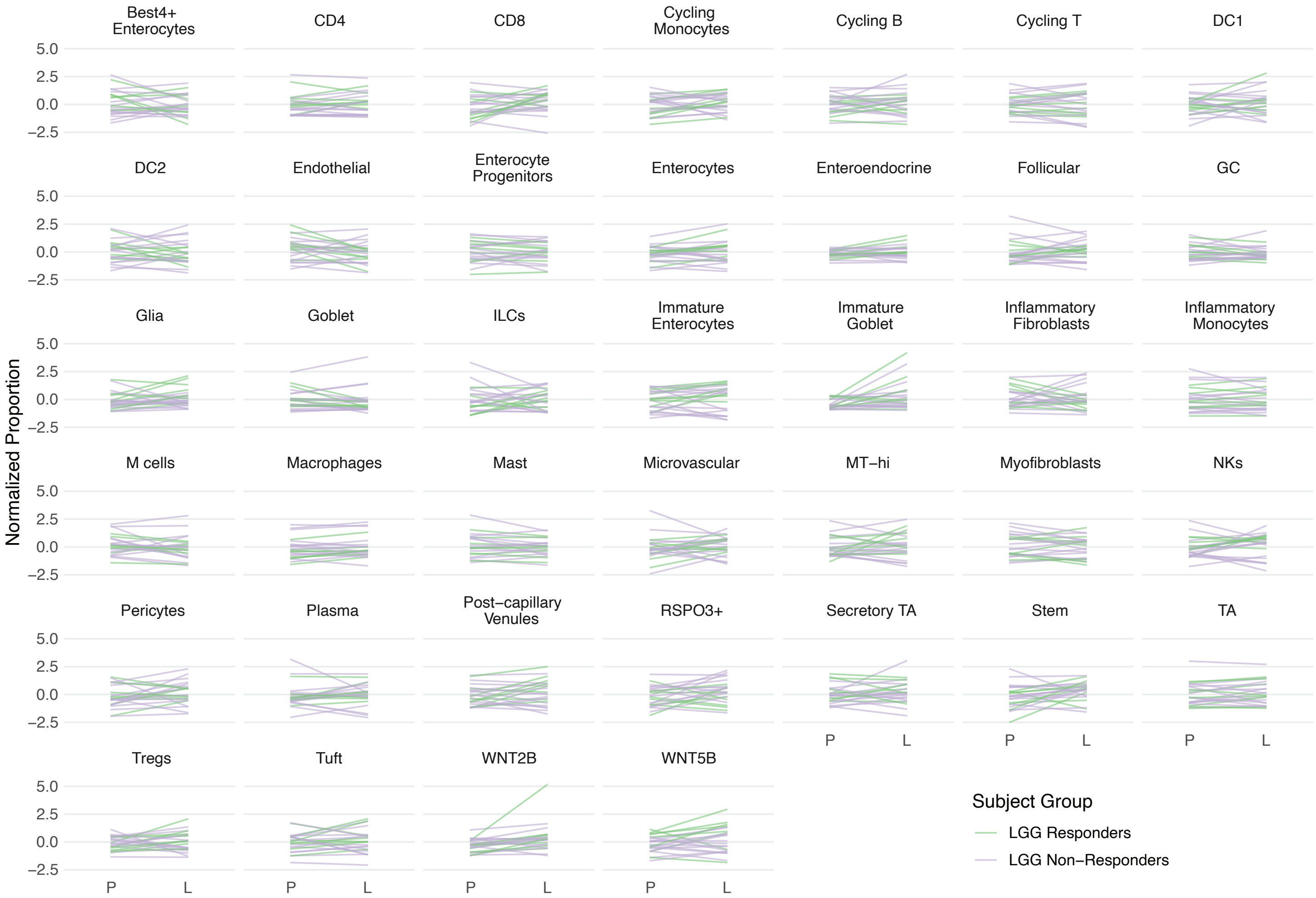

Normalized proportions of cell types (y axis) between treatments (placebo and LGG, x-axis). Subpanels show estimates for each cell type assessed in cell deconvolution analysis, based on tissue level RNA-seq data. X axis on subplots show treatment (P=placebo, L=LGG). Y axis on subplots shows estimated cell type proportions (rescaled to the mean and range of each individual cell type). Each line represents a single individual, where color indicates group (LGG responders in green and non-responders in pink). Lines that go up show an increase in cell proportion in a given subject after LGG exposure, and vice versa.

Figure S4

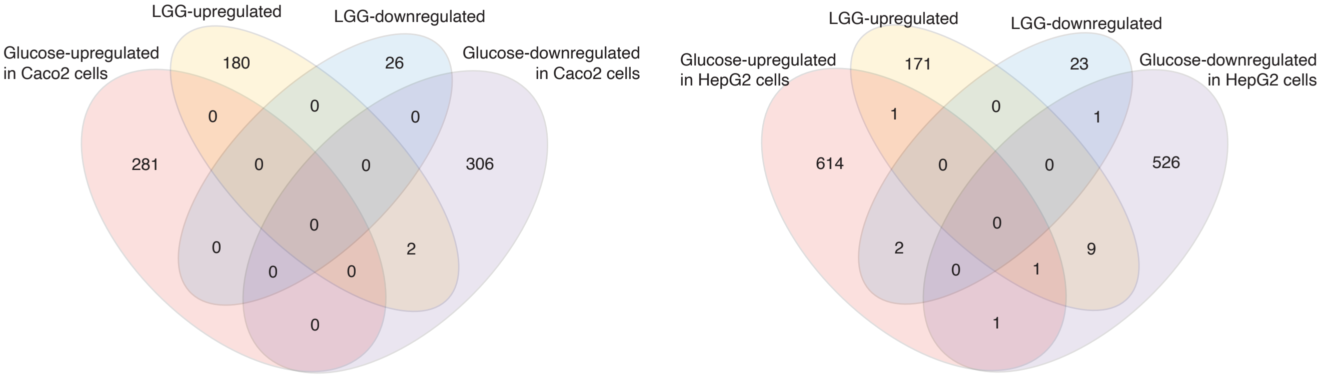

Overlap betweenLGG-up/down-regulated genes in jujunum and glucose-responding genes in Caco2 or Hep2G cells.

LGG-responding genes were defined as in main text (absolute log_2 fold change >0.5, FDR <0.05, LGG-responder group). For Caco2, gene lists provided by the authors (Boztepe and Gulec Genes & Nutrition (2018) 13:11) were used, and we then selected genes with an absolute log_2 fold change of >0.5 and FDR <0.05. For HepG2, we used the raw microarray data from Jeong et al PLoS One 2011;6(7):e22544 (GEO accession number GSE22074) and analyzed those with the GEO2R tool using standard settings to define glucoseregulated genes using the same cutoffs as above. Overlaps are visualized by 4-way Venn diagrams .
