# Supplementary material for: Personalized B cell response to the Lactobacillus rhamnosus GG probiotic in healthy human subjects: a randomized trial
Source: Gut Microbes. 2020 Dec 4;12(1):1854639. doi: 10.1080/19490976.2020.1854639 (PMC7722709; doi:10.1080/19490976.2020.1854639)
Supplement: Supplemental Material [file KGMI_A_1854639_SM3886.zip › Supplementary information/Supplementary Text 1 all.pdf]

Supplementary Text 1

CONSORT checklist	2
CONSORT flow diagram	5
STUDY PROTOCOL	6
Analysis of potential batch effects	43

CONSORT 2010 checklist of information to include when reporting a randomised trial*

Section/Topic	Item No	Checklist item	Reported on page No
**Title and abstract**			
	1a	Identification as a randomised trial in the title	1
	1b	Structured summary of trial design, methods, results, and conclusions (for specific guidance see CONSORT for abstracts)	2
**Introduction**			
Background and objectives	2a	Scientific background and explanation of rationale	3-5
	2b	Specific objectives or hypotheses	5
**Methods**			
Trial design	3a	Description of trial design (such as parallel, factorial) including allocation ratio	15-18
	3b	Important changes to methods after trial commencement (such as eligibility criteria), with reasons	NA
Participants	4a	Eligibility criteria for participants	15
	4b	Settings and locations where the data were collected	15-18
Interventions	5	The interventions for each group with sufficient details to allow replication, including how and when they were actually administered	15-18
Outcomes	6a	Completely defined pre-specified primary and secondary outcome measures, including how and when they were assessed	Not applicable, exploratory endpoint
	6b	Any changes to trial outcomes after the trial commenced, with reasons	Not applicable
Sample size	7a	How sample size was determined	Exploratory endpoint, page 15-18
	7b	When applicable, explanation of any interim analyses and stopping guidelines	Not applicable
Randomisation:			
Sequence generation	8a	Method used to generate the random allocation sequence (	16-17
	8b	Type of randomisation; details of any restriction (such as blocking and block size)	16-17
Allocation concealment	9	Mechanism used to implement the random allocation sequence (such as sequentially numbered containers), describing any steps taken to conceal the sequence until interventions were assigned	16-17

mechanism			
Implementation	10	Who generated the random allocation sequence, who enrolled participants, and who assigned participants to interventions	15-17
Blinding	11a	If done, who was blinded after assignment to interventions (for example, participants, care providers, those assessing outcomes) and how	NA
	11b	If relevant, description of the similarity of interventions	NA
Statistical methods	12a	Statistical methods used to compare groups for primary and secondary outcomes	19-20
	12b	Methods for additional analyses, such as subgroup analyses and adjusted analyses	7-10
**Results**			
Participant flow (a diagram is strongly recommended)	13a	For each group, the numbers of participants who were randomly assigned, received intended treatment, and were analysed for the primary outcome	15-17
	13b	For each group, losses and exclusions after randomisation, together with reasons	15-17
Recruitment	14a	Dates defining the periods of recruitment and follow-up	www.clinicaltrials.gov
	14b	Why the trial ended or was stopped	NA
Baseline data	15	A table showing baseline demographic and clinical characteristics for each group	24
Numbers analysed	16	For each group, number of participants (denominator) included in each analysis and whether the analysis was by original assigned groups	5-12, but exploratory endpoint
Outcomes and estimation	17a	For each primary and secondary outcome, results for each group, and the estimated effect size and its precision (such as 95% confidence interval)	5-12, but exploratory endpoint
	17b	For binary outcomes, presentation of both absolute and relative effect sizes is recommended	Not applicable
Ancillary analyses	18	Results of any other analyses performed, including subgroup analyses and adjusted analyses, distinguishing pre-specified from exploratory	5-12, but exploratory endpoint
Harms	19	All important harms or unintended effects in each group (for specific guidance see CONSORT for harms)	15-17
**Discussion**			
Limitations	20	Trial limitations, addressing sources of potential bias, imprecision, and, if relevant, multiplicity of analyses	12-14
Generalisability	21	Generalisability (external validity, applicability) of the trial findings	12-14
Interpretation	22	Interpretation consistent with results, balancing benefits and harms, and considering other relevant evidence	12-14
**Other information**			

Registration	23	Registration number and name of trial registry	16
Protocol	24	Where the full trial protocol can be accessed, if available	Submitted with manuscript
Funding	25	Sources of funding and other support (such as supply of drugs), role of funders	23

*We strongly recommend reading this statement in conjunction with the CONSORT 2010 Explanation and Elaboration for important clarifications on all the items. If relevant, we also recommend reading CONSORT extensions for cluster randomised trials, non-inferiority and equivalence trials, non-pharmacological treatments, herbal interventions, and pragmatic trials. Additional extensions are forthcoming: for those and for up to date references relevant to this checklist, see www.consort-statement.org.

CONSORT
TRANSPARENT REPORTING of TRIALS

CONSORT 2010 Flow Diagram

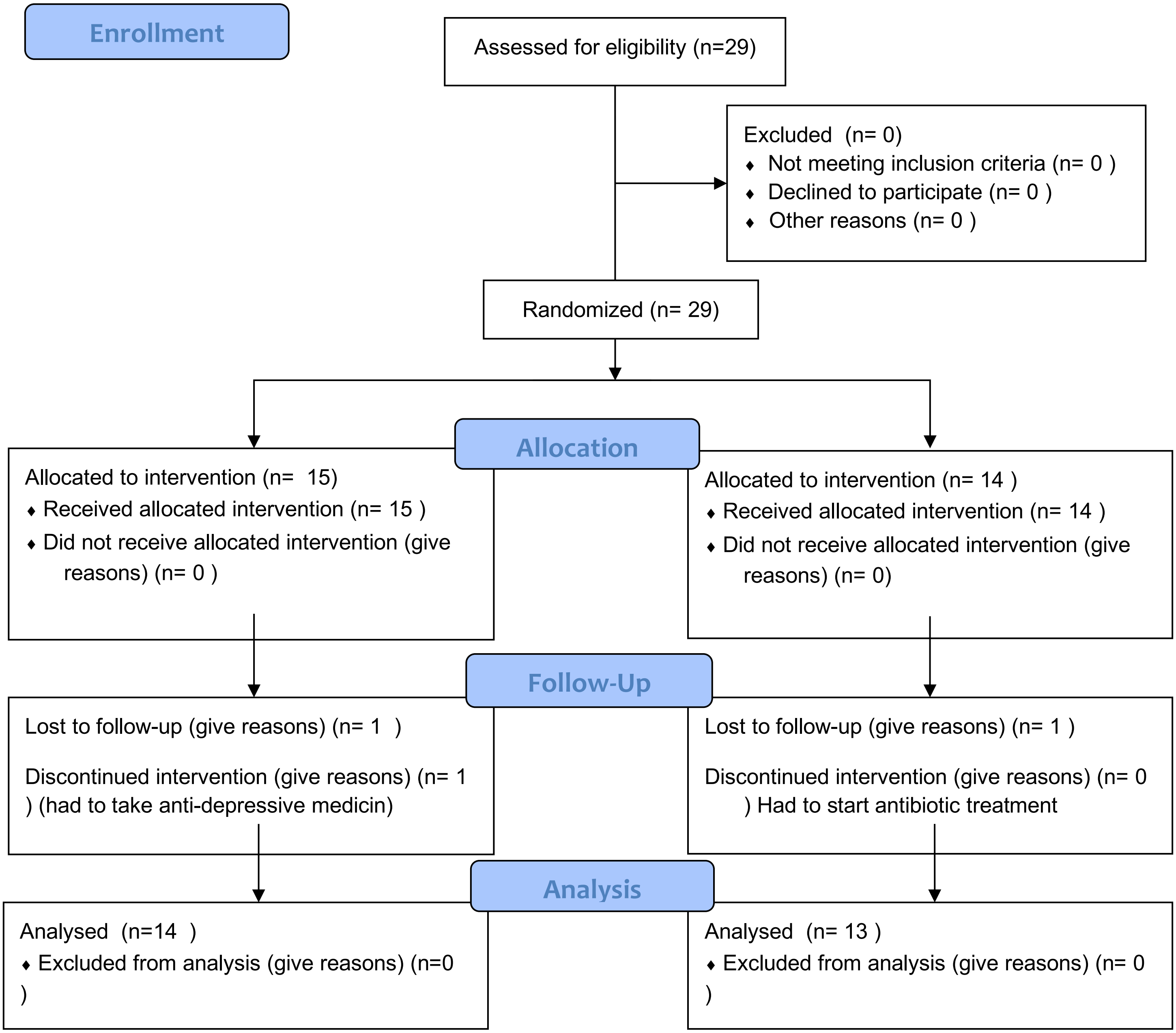

STUDY PROTOCOL

Defining the normal human response to probiotics and developing an in vitro system to identify new microbes with probiotic functions.

Study ID number:
56250
Chr. Hansen Internal number:
HND-GI-024

Version:
03

Date:
17-03-2017

Sponsor:
Chr. Hansen A/S
Bøge Allé 10-12
DK-2970 Hørsholm,
CVR:12516479

Principal Investigator:
Kim B. Jensen
Copenhagen University, BRIC
Ole Maaaløes Vej 5
2200 København N

This document is a confidential communication of the involved parties. The information may be provided only to investigators, appropriate study personnel, members of the Institutional Review Boards / Independent Ethics Committees and personnel of the authorities to whom it is submitted. No unpublished information contained herein [illegible]

Signatures and Agreement with Protocol

We, the undersigned, acknowledge that we have read this protocol.

We agree to conduct this study in accordance with the study protocol and the current version of the Declaration of Helsinki, and with any additional local laws and regulations.

Principal Investigator	*Date*

Assoc. Professor, PhD Kim B. Jensen

Copenhagen University, BRIC

Ole Maaaløes Vej 5

2200 København N

Clinical lead	*Date*

Dr. med Jakob Hendel

Gastroenheden

Herlev Hospital

Herlev Ringvej 75

2730 Herlev

Sponsor's representative	*Date*

Christa Broholm

Post Doc

Chr. Hansen A/S

Bøge Alle 10-12

Hørsholm

List of Responsible Parties

Chr. Hansen A/S Contact

Christa Broholm

Post Doc

Chr. Hansen A/S

Bøge Alle 10-12

Hørsholm

Lead Scientist

Assoc. Professor, PhD Kim B. Jensen

Copenhagen University, BRIC

Ole Maaaløes Vej 5

2200 København N

Operational Lead & clinical site

Dr. med Jakob Hendel

Gastroenheden

Herlev Hospital

Herlev Ringvej 75

2730 Herlev

Synopsis

Title of Study:

Defining the normal human response to probiotics and developing an in vitro system to identify new microbes with probiotic functions

Sponsor:

Chr. Hansen A/S

Principal Investigator:

Assoc. Professor, PhD Kim B. Jensen

Study Center:

Gastroenheden, Herlev Hospital

Planned Study Period:

6 weeks

Objectives:

1. to investigate the correlation between the in vivo response in gut biopsies and the in vitro response in isolated and cultured mini guts after administration of the probiotic strain *Lactobacillus rhamnosus* GG (LGG®).
2. to develop methods to culture and analyse probiotic effects on human mini guts in large scale screening platforms.

Methodology:

The study is a mono-center, randomized, semi-blinded, placebo-controlled, cross-over, proof-of principle study in healthy volunteers. The study will investigate whether it is possible to use cultures of human mini-guts to determine the probiotic effect of LGG, which is a known and clinically approved microbe.

The study includes 3 visits. The first visit serves to check inclusion/exclusion criteria/complete questionnaire/give information about the study and receive consent. At visit 2 and 3 the included individuals will drink a placebo blend or a drink containing 450 billion CFU Lactobacillus rhamnosus GG (LGG®) and they will subsequently be biopsied from the upper intestine during endoscopy while under nurse administered propofol sedation (NAPS). There will be four weeks between visit 2 and 3.

At visit 2 and 3, 6 biopsies will be taken from duodenum and 6 biopsies will be taken from jejunum (approximately 15mg each), luminal fluids will be collected (25 mL pr visit) and one blood sample (of 20mL pr visit) will be collected. At the visit where placebo blends are given, there will additionally be isolated intestinal stem cells to grow mini-guts in vitro and isolate bacterial strains from the luminal fluid. The concentration of LGG will be analysed in luminal fluid at both visit 2 and 3.

The down stream analysis will include a combination of expression analysis, microbiome analysis and classification of the individuals based on analysis of blood samples. The isolated mini-guts will be used to investigate the normal function of intestinal epithelial cells as well as study the effects of LGG and other microbes. The luminal fluid will be used to isolate microbes for commercialization at Chr. Hansen.

Planned Number of Subjects:

24

Main Criteria for Inclusion:

Healthy men and women with an age between 18 and 35 years and a BMI below 30. No medications allowed.

Test Product:

Lactobacillus is a genus of lactic acid producing, gram positive bacteria. Lactobacillus rhamnosus (LGG®) is the world's best documented probiotic strain and has been used in food and dietary supplements since 1990. The strain has been described in more than 760 scientific publications and studied in more than 260 clinical studies. The US Food and Drug Administration (FDA) have designated the LGG® strain as Generally Recognized as Safe (GRAS). The product has a minimum potency of 30 billion (3.0E+10) CFU (Colony Forming Units) per capsule at time of release.

Reference Product (placebo):

The placebo product is an identical blend except for the absence of probiotics.

Duration of Intervention:

The subjects will participate in two acute interventions including intake of probiotics/placebo and subsequent endoscopy altogether lasting approx. 3 hours.

Abbreviations and Definition of Terms

CFU	Colony forming units
CRF	Clinical report form
EFSA	European Food Safety Authority
FDA	Food and Drug Administration
GRAS	Generally Recognized as Safe
IEC	Independent Ethics Committee
IP	investigational product
IRB	Institutional Review Board
SAE	serious adverse event

Table of Contents

Signatures and Agreement with Protocol 2

List of Responsible Parties 3

Abbreviations and Definition of Terms 5

1. Ethics and Regulations 9
- 1.1 Independent Ethics Committee 9
- 1.2 Ethical Conduct of the Study 9
- 1.3 Ethical aspects 9
- 1.4 Subject Information and Informed Consent 9
- 1.5 Protocol Changes 9
- 1.6 Protocol Deviations 10
- 1.7 Insurance 10

2. Introduction 11
- 2.1 Background 11
- 2.2 Assessment of Anticipated Benefits and Risks 12
- 2.3 Additional Information 12

3. Study Objectives 13
- 3.1 Primary Objective 13
- 3.2 Secondary Objective 13

4 Investigational Plan 14
- 4.1 Overall Study Design 14
- 4.2 Discussion of Study Design 16
- 4.3 Recruitment and selection of study subjects 16
- 4.4 Inclusion Criteria 16
- 4.5 Exclusion Criteria 17
- 4.6 Subject Withdrawal Criteria 17

5. The Probiotic Product 19
- 5.2 Description of LGG 19
- 5.3 Selection of Dosages and Dosage Regimen 19
- 5.4 Other Restrictions and Instructions for Study Subjects 20

6. Description of Visits and Study Periods 21
- 6.1 Visit 1 (Screening Visit, Day -14) 21
- 6.2 Visit 2 and 3 (endoscopies on Day 0 and 28) 21

7. Laboratory Analyses and research approaches 21
- 7.1 Isolation of epithelium and stroma from biopsies 21
- 7.2 Culture of intestinal epithelium cells as mini-guts 21
- 7.3 Analysis of composition of luminal fluids 22
- 7.4 Screening for microbial-epithelial interactions in vitro 22
- 7.5 RNA analysis 22

7.6 Blood samples 23
7.7 SNPs 23
7.8 shipment, handling and storage of samples 23

8. Other Assessments 24

9. Data Management, statistical considerations and material handling 24
9.1 Data Collection and Processing 24
9.2 Confidentiality 24
9.3 Statistical considerations 24
9.4 Research biobank 25
9.5 Biobank for future research 25
9.6 Termination of Study 26

10. Side effects, risks, and disadvantages 27
10.1 expected side effects 27
10.2 Procedures for handling severe side effects 27
10.3 Reporting of Serious Adverse Events. 27
10.4 Follow-up of Adverse Events and Serious Adverse Events 28
10.5 Procedures in Case of Medical Emergency 28

11. Study Documentation 29
11.1 Study Files 29
11.2 Retention of Records 29
11.3 Publication of Results 29

12. Economy and financial compensations 30

References 32

Appendix 1 – Independent Ethics Committee 35

Appendix 2 – Study Administrative Structure 36

1. Ethics and Regulations

1.1 Independent Ethics Committee

The relevant Independent Ethics Committee (IEC) will be consulted about this study and the study initiated only after a favorable opinion has been obtained.

A report of the study will be sent to the IEC after the study.

The address of the IEC is included in Appendix 01 of this protocol.

1.2 Ethical Conduct of the Study

The study will be conducted in accordance with the ethical principles set forth in the current version of the Declaration of Helsinki, and all applicable local regulatory requirements.

1.3 Ethical aspects

The project is expected to result in limited risks, adverse effects and discomfort to the subjects. The clinical research will allow for the establishment of a methodology which in the future will enable the identification of microbes with beneficial effects for human health. The perspectives in the methodology are the identification of patient specific combinations of microbes, which will improve treatment options for patients with gastrointestinal disorders. The perspectives outweigh the risks and discomfort associated with the endoscopy, biopsies and blood samples. The study will be conducted in accordance with the ethical principles set forth in the current version of the Declaration of Helsinki.

1.4 Subject Information and Informed Consent

Written, informed consent will be obtained from all subjects prior to entry into the study. The Investigator / study personnel will explain verbally to each subject the objectives, nature, significance, risks and implications of the study before inclusion. This information will also be included in the written subject information sheet.

In particular, the subjects will be informed about the following:

- The possibility of withdrawing from the study at any time without loosing any benefits the subject is entitled to
- How personal and health-related data will be collected and used during the study

The subjects will be given time to discuss any questions and make a decision regarding participation in the study. All subjects will receive a copy of the subject information sheet and the signed informed consent form. The original will be retained by the Investigator.

1.5 Protocol Changes

Substantial amendments to this protocol may be implemented only after a favorable opinion of the independent ethical committee has been obtained. Amendments to the protocol are regarded as substantial if they have a significant impact on

- The safety, physical health and mental integrity of the study subjects
- The scientific value of the study
- The conduct or the management of the study

- The quality or safety of the probiotic used in the study

Any amendments to this protocol will be signed by the signatories included in section 1.

If an event occurs related to the conduct of the study or the development of the test product which may affect the safety of the study subjects, the sponsor and the study investigator may take appropriate measures to protect the subjects against immediate hazards. The Sponsor or the Sponsor's representative will inform the applicable IEC/IRB authorities of the new events and the measures taken as soon as possible.

1.6 Protocol Deviations

No systematic deviations from the protocol are allowed, and no protocol waivers will be given. All protocol deviations noted during the study (whether by the Investigator or the Sponsor) will be recorded and evaluated as major or minor.

1.7 Insurance

All subjects will be insured by the Sponsor (Chr. Hansen) against potential damage caused by intake of Lactobacillus LGG. All injuries which are to be attributed to the biopsies are covered by the patient insurance according to local legal requirements in Denmark where the study takes place.

2. Introduction

2.1 Background

Probiotics are defined as ‘live microorganisms which when administered in adequate amounts, confer a health benefit to the host’ (FAO/WHO, 2001). Such microbes (typically bacteria) are present in certain fermented foods, added to dairy foods such as yoghurts, fermented milk or in oral dietary supplements. As an example, probiotics added to yoghurt help restore the bacterial flora after antibiotics treatment and may boost the immune system and alleviate minor intestinal problems. Recently, the important role of microbes in many important diseases and syndromes has been demonstrated, for example in metabolic and gastrointestinal disorders, and diseases such as autism (Flint et al., 2012; Hsiao et al., 2013). Moreover, it is increasingly clear that the composition of commensal microbiome dictates susceptibility and response to pathogens as demonstrated by faecal transplantation experiments (Li et al., 2016). This has generated an even higher interest in the function of microbes, and an increased push to identify new beneficial microbes that will become the next generation of probiotics. For the first time such probiotics will be truly interfacing food science and medicine.

Microbiota with probiotic properties influence our gastrointestinal system using a number of different mechanisms. Certain microbes lyse as soon as they reach the acidic environment in the stomach and resulting debris influence the gut via interaction with pattern recognition receptors of the TLR and NOD family (Douillard et al., 2013). Other bacteria, like BB-12, survive the harsh conditions of the upper gastrointestinal tract, and probiotic benefits are in these cases very likely stimulated via secretion of messengers or interactions with intestinal epithelial cells (Jungersen et al., 2014). The effects on the epithelial cells are diverse and can involve enhanced barrier formation via stabilisation of tight junctions (Ulluwishewa et al., 2011), altered proliferation (Canonici et al., 2012; Di Giancamillo et al., 2008; Sakata et al., 1999), and elevated expression of pro-inflammatory cytokines (Lebeer et al., 2012). No single methodology currently enables anyone to capture all known and unknown effects of microbiota on the epithelium in a high-throughput manner.

Current probiotics discovery is based on empirical testing on a single human cancer cell line (CACO-2) or in mouse/rat models. While human cancer cells are easy to work with, their similarity to cells in a functional human gut is exceedingly low, and they fail to recapitulate the complexity of the human gut, and they are representative of only small part of the gut. Animal studies have the advantage of relying on a physiological context, but are unsuitable as a discovery platform due to the high costs, which limits the number of possible trials. Animal experiments also raise ethical concerns for the industry. Moreover, mice and rats are physiologically different from humans. Alternative models of human gut are therefore required to enable us to identify new probiotics based on scientific merits.

In the present study we use human intestinal gut stem cells collected from healthy human guts. Based on a defined cell culture system, which has been optimised for the last 3 years in the Jensen group, we can grow these reproducibly in the laboratory from essentially any individual, where they recapitulate the normal gut structure. We call these structures ‘mini-guts’. The mini-guts can be frozen down, remain viable upon thawing and can be expanded unlimitedly for in vitro screening purposes. Moreover, the bowel can be subdivided into at least seven distinct regions (duodenum, jejunum and ilium, which are parts of the small bowel, and ascending, transverse and descending colon and the rectum, which constitute the colon), with specialised functions. Each of these regions can be maintained in vitro as distinct mini-guts (Middendorp et al., 2014; Wang et al., 2015). Seminal experiments using mouse mini-guts have shown that they can elicit an appropriate cellular response in vitro when exposed to purified strain of bacteria (Lukovac et al., 2014). Based on the above it is

possible to establish a unique collection of material representing the entire lower gastrointestinal tract from both normal donors and patients with specific disorders. Such a collection will form the basis for identifying microbes with probiotics effects, which will benefit the majority or specific subsets of the human population, something that no other technology can do.

2.2 Assessment of Anticipated Benefits and Risks

The project is, as described above, expected to result in limited risks, adverse effects and discomfort to the subjects. The clinical research will allow for the establishment of a methodology which in the future will enable the identification of microbes with beneficial effects for human health. The perspectives in the methodology are the identification of patient specific combinations of microbes, which will improve treatment options for patients with gastrointestinal disorders. The perspectives outweigh the risks and discomfort associated with the endoscopy, biopsies and blood samples.

2.3 Additional Information

The Investigator should refer to Product Information provided by the Sponsor for additional information regarding the probiotic LGG.

3. Study Objectives

The objective of the project is to develop a human in vitro system using mini-guts to screen for health beneficial probiotics on a large scale. The development of a relevant human in vitro system will allow us to:

i. Screen lead candidates, and move directly into human studies, which will dramatically speed up the development of next generation probiotics and minimize use of animal models significantly.
ii. Scientifically document the effects of probiotics.
iii. Establish a human quality control (QC) assay for the production of probiotics.

3.1 Primary Objective

Primary objective is to investigate the correlation between the in vivo response in gut biopsies and the in vitro response in isolated and cultured mini guts after administration of the probiotic strain *Lactobacillus rhamnosus* GG (LGG®).

3.2 Secondary Objective

Secondary objective is to develop methods to culture and analyse probiotic effects on human mini guts in large scale screening platforms

4 Investigational Plan

4.1 Overall Study Design

This study is a mono-center, randomized, semi-blinded, placebo-controlled, cross-over, proof-of principle study in healthy volunteers. The study will investigate whether it is possible to use cultures of human mini-guts to determine the probiotic effect of LGG, which is a known and clinically approved microbe.

The study includes 3 visits. The first visit serves to check inclusion/exclusion criteria/complete questionnaire/give over information about the study and receive consent. At visit 2 and 3 the included individuals will be biopsied from the upper intestine during endoscopy while under nurse administered propofol sedation (NAPS) with four weeks between each (Figure 1); once drinking a placebo blend and once drinking a blend containing 450 billion CFU Lactobacillus rhamnosus GG (LGG®).

At visit 2 and 3, 6 biopsies will be taken from duodenum and 6 biopsies will be taken from jejunum (approximately 15mg each), luminal fluids will be extracted (25 mL pr visit) and one blood sample (of 20mL pr visit) will be collected. At the visit where placebo capsules are given, there will additionally be isolated intestinal stem cells to grow mini-guts in vitro and isolate bacterial strains from the luminal fluid.

The down stream analysis will include a combination of expression analysis, microbiome analysis and classification of the individuals based on analysis of blood samples. The isolated and cultured mini-guts will serve as a model to understand the normal function of intestinal epithelial cells and study LGG and other microbes. The luminal fluid will be used to isolate microbes for commercialization at Chr. Hansen.

Figure 1 Study overview

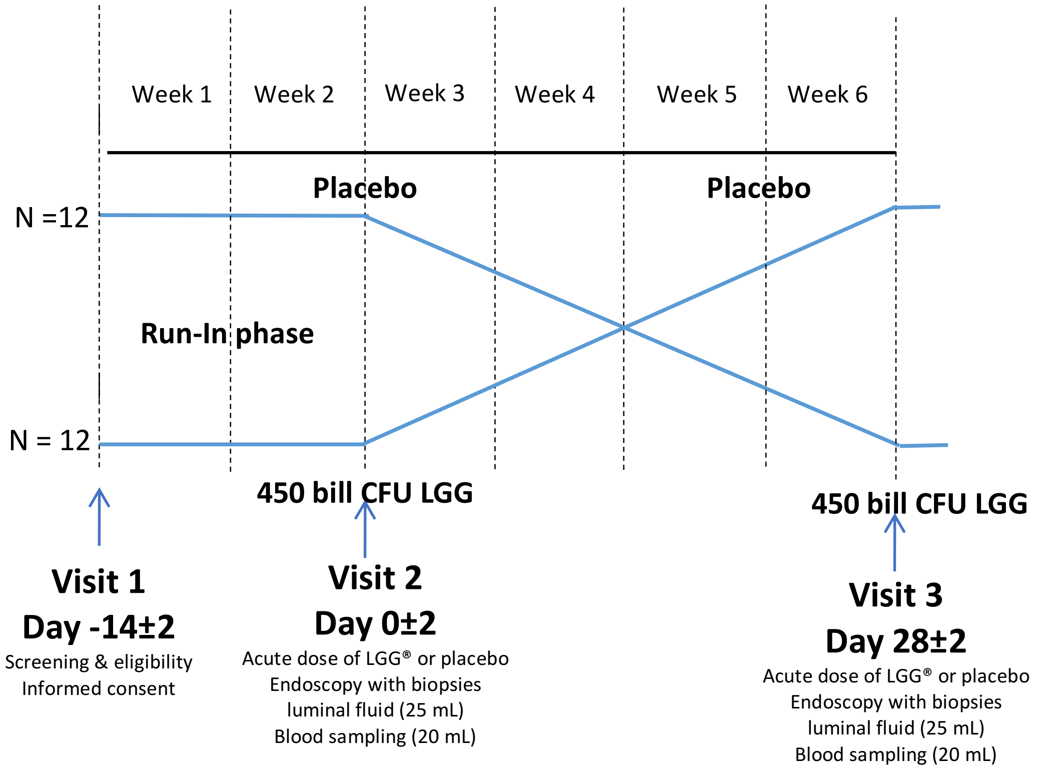

Table 1 Study flow chart

**Visit**	**1**	**2**	**3**
Day	-14±2	0±2	28±2
Check of inclusion/exclusion criteria	X	X	X
Written informed consent	X		
Demographic data	X		
Smoking habits and alcohol consumption	X		
Relevant medical history	X		
Check for concomitant medication	X	X	X
Complete physical examination	X		
Height, body weight and BMI	X		
Biopsy under propofol sedation (NAPS)		X	X
Blood samples (20 mL per visit)		X	X
Luminal fluid (25 ml pr visit)		x	x
Intake of LGG or Placebo		X	X

4.2 Discussion of Study Design

A run-in period is included to wash out the potential impact of any pre-study probiotic products. The four weeks between visit 2 and 3 serves to give the intestinal tissue time to heal between biopsies. The dose (450 billion CFU) and timing of the product was based on the study by (van Baarlen et al. 2011), who investigated the acute effect of LGG on intestinal tissue. A placebo blend, which contains the same as the blend containing the probiotic (primarily carbohydrates), is included to ascertain that the effect observed on the intestinal tissue can be ascribed to the probiotic alone as it could be speculated that carbohydrates even at low doses affect intestinal cells in the fasted state. A cross over design was chosen to obtain the statistical benefits of the subjects being their own control.

4.3 Recruitment and selection of study subjects

The study population should reflect the general, European adult population. As only a small fraction of the general European population and thereby the study population are diseased, only healthy individuals will be included.

Gastroenhedens Ambulatorium, Herlev Hospital will by local advertisement and www.forsøgsperson.dk recruit healthy volunteers to the study. As part of the recruitment to the project the individuals will receive written information related to their participation in the project.

Participation in the project is on completely voluntarily basis after oral and written information in concordance with the Helsinki V-declaration. The volunteers can have a companion at the conversation, where information is received and will have the opportunity to discuss the project with a companion or will be able to contact one of the project leaders for additional information, and alternatively an additional conversation related to project inclusion.

The oral information will be given by one of the project partners in a quiet room at the Gastrounit, Herlev Hospital. During the conversation it will be possible to read the provided written information, receive oral information and ask probing questions. Accordingly, there is a clear connection between the information given to the individual and call for patient consent, although patients will have up to 24 hours to consider the information given and can at any point withdraw their consent.

The individual will during the conversation be given the latest version of ”Forsøgspersoners rettigheder i et sundhedsvidenskabeligt forskningsprojekt” from ‘Den Nationale Videnskabsetiske Komite’ august 2014.

4.4 Inclusion Criteria

Subjects should fulfill the below listed inclusion criteria to be eligible for the study:

1. Healthy men or women
2. No medication
3. Age between 18 and 35 years
4. BMI below 30
5. Provided voluntary written informed consent

4.5 Exclusion Criteria

Subjects meeting one or more of the below listed exclusion criteria can not be enrolled in the study:

1. All clinical diagnoses and disorders requiring medicine
2. Patient diagnosed with inflammatory bowel disease such as ulcerative colitis and Crohn's disease, colorectal cancer or suffering from irritable bowel syndrome.
3. Pregnant and women who are breast-feeding
4. Patient with known blood clothing disorders
5. Patients with clinical psychiatric diagnoses (including dementia)
6. Individuals who have undergone abdominal surgery, which might have effect on the GI function, except appendectomy and cholecystectomy
7. Individuals with high blood pressure (≥140 mmHg /90 mmHg)
8. Systemic use of antibiotics or steroids or antimicrobial medication in the last 4 months
9. Daily usage of NSAID in the last 2 months or incidental use in the last 2 weeks prior to screening
10. Usage of medications, except oral contraceptives, during the 14 days prior to screening
11. Lactose intolerance
12. Participation in other clinical trials in the past three months
13. Regular use of probiotics in the last 6 weeks
14. Smoking
15. Planned changes to current diet or exercise regime
16. Use of laxatives, anti-diarrheals, anti-cholinergics within last 4 weeks prior to screening
17. Use of immunosuppressant drugs within last 4 weeks prior to screening
18. Ulcer or malignancy in the intestine which is discovered during second visit

4.6 Subject Withdrawal Criteria

Participation in the study is voluntary, and subjects have the right to withdraw from the study at any time without providing a reason. If a study subject chooses to withdraw, the study personnel must be informed, and the reason for discontinuation should be documented in the case report form.

The Investigator has the right to terminate participation of any subject at any time if they deem it in the subject's best interest.

Examples of possible reasons for premature withdrawal of a study subject include:

- Subject withdraws consent for personal reasons
- Subject's general condition contraindicates continuing the study, as judged by the study personnel or the medical expert

- Significant non-compliance with study protocol or lack of cooperation
- Serious Adverse Event (SAE)
- Lost to follow-up
- Other reasons as determined by the Investigator
- No reason given by subject

5.2 Description of LGG

Lactobacillus is a genus of lactic acid producing, gram positive bacteria. Lactobacillus rhamnosus (LGG®) is a well-documented probiotic strain and has been used in food and dietary supplements since 1990. The strain has been described in more than 760 scientific publications and studied in more than 260 clinical studies. The US Food and Drug Administration (FDA) have designated the LGG® strain as Generally Recognized as Safe (GRAS). In Europe, Lactobacillus rhamnosus (LGG®) has been granted Qualified Presumption of Safety (QPS) status since 2007 by the European Food Safety Authority (EFSA) – a status granted on species level.

The investigational product is vegetable capsule containing LGG (Table 2). The product has a minimum potency of 30 billion (3.0E+10) CFU (Colony Forming Units) per capsule at time of release, and a minimum potency 3 billion CFU per capsule by end of shelf life. The placebo product is an identical capsule except for the absence of probiotics.

The products will be produced under GMP conditions at Chr. Hansen laboratory in Hørsholm, Denmark, which is certified for food production.

Table 2 Composition of investigational product

	**Probiotic capsules lactobacillus**	**Placebo capsules**
Manufacturing	Chr. Hansen A/S, Denmark	Chr. Hansen A/S, Denmark
Brief description	Probio-Tec® LGG® VCap-30 Bli V2 are size 1, opaque, hard hypromellose capsules, each containing 270 mg of a standardized white to light beige fine powder.	Placebo capsules are size 1, opaque, hard hypromellose capsules, each containing 270 mg of a standardized white to light beige finepowder.
Weight & Size	Size 1 HPMC capsules	Size 1 HPMC capsules
Capsules shell	Hypromellose, titanium dioxide	Hypromellose, titanium dioxide
Active Ingredients	Lactobacillus rhamnosus LGG®	None
Supplied as	1 capsules per CSP Active vial<br>Packaged in a protective atmosphere	1 capsules per CSP Active vial<br>Packaged in a protective atmosphere
Expiry date	31 October 2018	31 October 2018
Storage conditions	Store at 2 - 8 °C	Store at 2 - 8 °C

The study products will be provided as an acute dosing for all subjects at Visit 2 and Visit 3 according to randomization number. The blend of the capsules will be dissolved in 50 ml water before ingestion.

5.3 Selection of Dosages and Dosage Regimen

The blend from 15 capsules with or without 30 billion CFU LGG will be mixed with 50 ml water and swallowed 2 hours before endoscopy and biopsying procedures. The dosing and timing is based on a

previous study by (van Baarlen et al.), which showed an overall significant effect on gene expression in intestinal biopsies using a high (450 billion CFU) acute dose of LGG.

5.4 Other Restrictions and Instructions for Study Subjects

Subjects are not allowed to eat the dairy products shown in appendix 3 two weeks prior to visit 2 and during study period. In addition, subjects should abstain from all types of medicine, probiotics, vitamins, minerals and other food supplements as well as maintain normal lifestyle and dietary habits. We encourage subjects not to travel in the study period to avoid diarrhea and to abstain from alcohol intake two days prior to visit 2 and 3.

6. Description of Visits and Study Periods

6.1 Visit 1 (Screening Visit, Day -14)

At visit 1 the following procedures and assessments will be performed

- Subjects will receive oral and written information about the study and be allowed to ask questions
- Subjects will sign the informed consent document
- Inclusion and exclusion criteria will be reviewed
- Medical history will be collected
- Demographic data will be collected

6.2 Visit 2 and 3 (endoscopies on Day 0 and 28)

At visit 2 and 3 the following procedures and assessments will be performed

- Dispensing of probiotic/placebo product 2 hours before endoscopy
- Collection of fasting blood samples
- NAPS
- Endoscopy and biopsies
- Collection of luminal fluid
- Observation 30 minutes
- At visit 2, subjects receive instruction for next 4 weeks and new appointment time

7. Laboratory Analyses and research approaches

7.1 Isolation of epithelium and stroma from biopsies

The biopsies will following isolation from the individuals be separated into stroma and intestinal epithelium using methods relying on either proteinases or chelation. The isolated tissue will subsequently be processed for RNA isolation and for in vitro cultures using established culture conditions.

7.2 Culture of intestinal epithelium cells as mini-guts

In vitro cultures will be established from isolated epithelium using established conditions. Briefly, current conditions rely on seeding epithelial cells in matrigel or a similar extracellular matrix, and supplemented with medium containing Wnt3a, R-Spondin, Noggin and EGF as well as inhibitors of the p38-MAPK and TGFBR {Sato, 2011;Jung, 2011}. Under these conditions, differentiation is suppressed and cells are primarily proliferating. These conditions will be used to expand mini-guts from healthy individuals for subsequent analysis. In order to assess the effect on differentiated cells, cells will under certain circumstances be cultured as a confluent cell layer on plastic or be induced to differentiate as mini-guts using specific conditions {Jung, 2011}.

7.3 Analysis of composition of luminal fluids

It is possible to characterise the composition of the intestinal microbiome by sequence analysis of luminal fluids. Specifically, the microbial DNA is isolated from the fluids and full genome sequencing including the 16S ribosomal RNA will be performed by next generation sequencing methods using e.g. an Illumina platform. The composition is subsequently characterised by alignment to the archived sequence information for the different microbial phylogenies.

A further aim is to isolate and characterize microorganisms from intestinal fluid. To do this, samples will either be collected and frozen after optional addition of preservation additive for later processing or processed immediately. To isolate bacteria, samples will be serially diluted, inoculated into cultivation media, and growth of cultures monitored over time. Isolated strains will be screened for their ability to grow in various media, checked for purity, and their potential to improve food and health assessed.

7.4 Screening for microbial-epithelial interactions in vitro

Microbes are known to interact with cells in our body and support either proliferation, differentiation or cell death. In order to determine the effect of selected or combinations of microbes with epithelial cells we will mix the two either in solution, in a 3D gel or on plastic. We will follow the interactions using a number of techniques including but not limited to live imaging, RNA analysis to monitor dynamic changes in RNA expression of selected targets or via genome wide expression profiles, measuring permeability, and secretion of specific second messenger from the epithelium.

7.5 RNA analysis

Total RNA will be isolated from the patient specimens or from cultured mini-guts. RNA-seq libraries will be prepared using state of the art methods from commercial suppliers. Via specific enrichments in the preparation steps, the analysis will mainly focus on global expression analysis of protein-coding and long non-coding genes limiting our analysis to the subset of genes that are expressed at any given time in the cells of interests. Sequenced reads will be mapped to human genome (GRCh37/hg19) using canonical RNA-seq mapping and quantification tools such as tophat (https://ccb.jhu.edu/software/tophat/index.shtml) and cufflinks (http://cole-trapnell-lab.github.io/cufflinks) etc. Follow-up analyses will focus on measurable gene or isoform expression upon available sample conditions with the aim of finding LGG-based transcriptomic responses as well as similarities between organoids and their origins. A similar example of our intended RNA-seq analysis can be seen here (Yun et al., 2016). The analysis pipeline is not intended for identification of mutations and is not sufficient to determine germline mutations. Nonetheless, there is a small theoretical chance for randomly finding a known disease-related mutation. If our analyses indicate a serious disease-related mutation, the individual will be subject to genetic counselling, unless the individual unequivocally has indicated in the consent form that he/she is not interested obtaining this information.

7.6 Blood samples

HbA1c, CRP and hsCRP will be analysed immediately at Herlev hospital. In addition, plasma will be saved for analysis of cytokines and GLP-1 analogues, serum will be saved for analysis of iFABP and zonolin and PAX gen tubes will be prepared for RNA analysis.

7.7 SNPs

Genotyping will be done using Illumina SNP arrays as is standard in most genotyping studies. Specifically, DNA will be extracted and applied to the recent Illumina Omni 5M chip which covers 5M SNPs, including both non-coding and exome SNPs. Samples will be anonymised within analysis.

7.8 shipment, handling and storage of samples

- Two blood sample tubes will be sent directly to the biochemical laboratory at Herlev hospital for immediate analysis. The rest will be saved at -20 degrees for later analysis
- Intestinal tissue will be placed in ice-cold buffer or liquid nitrogen and transported directly to BRIC, University of Copenhagen by car. Isolated cell cultures will be stored in a liquid nitrogen tank and extracted RNA will be stored at – 80 degrees.
- Luminal fluid will be transported in an anaerobic jar to Chr. Hansen A/S.

8. Other Assessments

Medical History

At visit 1, the medical history will be recorded. Information about any disease is of relevance. This will be logged in the CFR form.

Physical Examination

At visit 1, a urine pregnancy test will be performed in female participants and height, weight, resting pulse and blood pressure will be noted for all participants. At visit 2 and 3 there will be performed a physical examination in relation to the endoscopy including blood pressure, heart rate, and breathing frequency.

Baseline Demographics

Baseline demographics will be collected at visit 1. This includes date of birth, gender and race/ethnicity

9. Data Management, statistical considerations and material handling

9.1 Data Collection and Processing

Data is collected as paper or electronic files, and entered into a project database via double-data entry.

9.2 Confidentiality

In order to maintain anonymity, subjects will only be identified by their initials and an assigned subject number for all documents submitted to the Sponsor. Documents that will not be submitted to the Sponsor and that identify the subject (such as the signed informed consent document) must be maintained in strict confidence by the Investigator.

9.3 Statistical considerations

The purpose of this protocol is to address whether intestinal epithelial cells grown in vitro as mini-guts/organoids will elicit an appropriate biological response when exposed to microbes in vitro. To pursue this aim we will perform two parallel statistical comparisons investigating: 1) the effect of LGG on genome-wide gene expression in intestinal tissue in vivo, and 2) the effect of LGG on genome-wide gene expression in mini-guts cultured in vitro.

Power studies using high-throughput sequencing are recognized as extremely challenging, due to that the variance is never known until the end of the experiment, and cannot be assessed in advance. It is also the case that with a globe RNA sequencing approach, the number of significant gene changes will increase with the number of subjects, simply because

it is harder to assess statistical significance with lowly expressed samples. In other words, saturation may never be realistically reached: the question is more whether one has enough samples to see the most important changes.

Therefore, number of samples has to be selected based on previous studies, or studies that are somewhat similar. It is important to note that our experiment is an unprecedented approach to investigate probiotics, and it is, therefore, difficult to extract variation data from previous studies. Indeed, no previous studies have reported effects of probiotics in human mini-guts, and only a few studies have examined the effects of probiotics on human intestinal cells in vivo. The Sandelin group (the statistics/genomics part of the consortium for this study) has recently made a study in both intestinal tissue and organoids in inflamed/non-inflamed contexts using a different RNA-based method (Boyd et al, in submission). This is not exactly the same as this study, but is informative of how many subjects that is necessary when working with complex tissue. This showed that having 20-25 human subjects is necessary in order to see robust responses – in this case finding in the order of thousands of genes that are significantly changing between groups after multiple testing correction (FDR<0.05, EdgeR general linear models using quasi-likelihood framework testing). We therefore choose 24 subjects.

9.4 Research biobank

A research biobank will be established in connection with the current project. The purpose of the research biobank is to 1) investigate the response of LGG and other microbes on cultured intestinal cell-lines (the mini-guts), and 2) to understand the normal function of intestinal epithelial cells, and 3) to isolate novel bacteria from the luminal fluid for commercialization at Chr. Hansen. The biobank will expire 31 Oct 2019. The mini-guts will be established from intestinal tissue. Mini-gut cultures (approx. 5 mill cells) and blood samples (approx. 40ml) will be stored behind lock at BRIC, Copenhagen University until the end of the study, whereas luminal fluid (approx. 50ml) will be stored behind lock at Chr. Hansen. All samples will be coded. The biological material will not leave Denmark.

After the expiration of the biobank remaining material will be transferred to a biobank at Chr. Hansen (see below)

9.5 Biobank for future research

The participants will be asked to sign a separate consent form to allow transfer of their biological material to a biobank at Chr. Hansen after this project is finished. The biobank will be reported to the Danish Data Protection Agency. Researchers interested in using the cells, blood samples or luminal fluid for other purposes than described in this protocol will always need to obtain approval from a national ethics committee.

9.6 Termination of Study

The sponsor reserves the right to terminate the study at any time. Conditions that may warrant termination of a study site or the entire study may include, but are not limited to, the following:

- Failure of the study centre to conduct the study in accordance with the protocol or any other local regulations.
- Failure of the study centre to enroll subjects at an acceptable rate.
- Failure of the study centre to ensure the quality of the data collected.
- New information on the study product, at any moment during the study, causing doubt about the benefit/risk ratio.
- A decision at the discretion of the Sponsor to discontinue the study for any reason.

10. Side effects, risks, and disadvantages

10.1 expected side effects

Blood samples: The patient will not experience significant side effects from the blood sampling (ca. 20mL). The needle mark from the blood sampling can give rise to soreness and maybe bruising.
In rare cases, the blood sampling can cause inflammation.
Intestinal biopsies and luminal fluid: The biopsies and luminal fluid will be obtained from healthy individuals who volunteer to be part of the project. Importantly these individuals will not have any indications of problems with the bowel. The subject may experience discomfort from the endoscopy examination, where the endoscope will start from the oral cavity and be transferred through the oesophagus via the stomach to the upper part of the small intestine. The procedure will be performed under NAPS and is expected to last no longer than 30 minutes per individual. Rarely (< 1 promille) individuals can experience bleeding following the endoscopy examination. If the bleeding does not stop after 15 minutes, can endoscopy directed blood coagulation be applied.
Ingestion of LGG: There are no known or expected risks or side effects for the product, but the risk of side effects cannot be excluded. Side effects, if any, are however expected to be minor and transient in nature. Side effects could typically be symptoms of digestive discomfort (abdominal discomfort, bloating, flatulence/passage of gas, borborygmi/rumbling stomach).
There are no known risks with administration of probiotics unless the study population consists of severely ill or immunocompromised patients. The strain, *lactobacillus* has been administered in a human intervention study many times. In Europe strains of lactobacillus belonging to the species have been granted Qualified Perceived as Safe (QPS) status by the European Food Safety Authority (EFSA). This means that strains of these species – including the strain used in this trial - is considered safe to use in food and as food supplements (EFSA, 2013).
Anaesthesia
Common side effects to Propofol-administration are burning/stinging, pain at the injection site (>10%), nausea, vomiting, headache after sedation stops (1-10%). Upon induction there is a risk of apnea, hypotension, bradycardia. Other rare risks include epilepsy-like seizures (<0.01-0.1%), pancreatitis, lung edema, loss of consciousness, hyper sexuality, anaphylactic reaction (<0.01%)

10.2 Procedures for handling severe side effects

A serious adverse event (SAE) is any untoward medical occurrence that at any dose:

- Results in death.
- Is life-threatening at the time of the event.
- Requires inpatient hospitalization.
- Results in persistent or significant disability or incapacity.
- Is another important medical event.

10.3 Reporting of Serious Adverse Events.

If a SAE occurs, study personnel should fill in a “Serious Adverse Event reporting form” and notify the Investigator. The Investigator should report all SAEs (whether or not considered related to study treatment) to the Sponsor within 24 h after awareness of the SAE. The reporting by the investigator to the Sponsor should cover the seriousness criteria, intensity and initial causality assessment.

The initial report of a SAE should as far as possible be supplemented by detailed information on diagnosis/symptoms, the relationship with the start of treatment and the latest dose of study treatment taken and any further relevant data. A follow-up report of the SAE will be written as applicable and sent to the Sponsor.

The Investigator shall report to the applicable Regulatory Authorities and Ethics Committee all relevant information about Suspected Unexpected Serious Adverse Reactions, in any case no later than seven days after knowledge by the Sponsor of a case which caused death or was life-threatening; no later than 15 days after knowledge for other unexpected serious adverse reactions.

The Sponsor will answer any complementary request made by the Health Authorities regarding any such event.

10.4 Follow-up of Adverse Events and Serious Adverse Events

After an SAE, the subject should be followed by any clinical or biological examination, considered as necessary by the medical judgment of the Investigator, until the SAE has resolved, stabilized, the investigator deems further observations or examinations to be no longer medically indicated or until the subject is under professional medical care. Follow-up should always be performed until a potential causality between the study treatment and the AE has been assessed.

10.5 Procedures in Case of Medical Emergency

In case of medical emergency, the clinical lead, MD Jakob Hendel, will be contacted.

11. Study Documentation

11.1 Study Files

The final protocol should be kept on-site in a dedicated investigator's study file, which should be kept strictly confidential. This file should also contain subject accountability records (screening and randomization logs), IP accountability records (dispensing), Sponsor/Investigator correspondence, IRB/IEC correspondence, protocol deviations, biological samples records, and SAE/Safety reports.

The investigator will keep a list of the subjects, identifying the names (with addresses and/or medical dossier numbers), their respective code number and the dates of start and end of the study, in order to verify the concordance between the data contained in the CRFs and that in the source documents.

All source documentation (i.e. subject diaries, medical notes, lab reports, electrocardiograms, etc.) should be available at the site.

11.2 Retention of Records

No study documents will be destroyed or moved to a new location without prior written approval from the Sponsor. If the Investigator relocates, retires, or withdraws from the clinical study for any reason, all records required to be maintained for the study should be transferred to an agreed-upon designee, such as another Investigator at the institution where the study was conducted.

11.3 Publication of Results

After completion of the study, the results will be tabulated, evaluated and issued as a complete final clinical study report. A summary of the report will be sent to the IEC if applicable.

All research results, whether negative, positive or inconclusive, will be published as quickly as possible in an international journal. Authorship can only be given to Investigators who fulfill the "Uniform Requirements for Manuscripts Submitted to Biomedical Journals" from the International Committee of Medical Journal Editors. Investigators, who do not meet these criteria will be acknowledged in a publication, if they so wish.

The trial will be registered at www.clinicaltrials.gov before enrolling subjects.

12. Economy and financial compensations

Subjects will be paid a fee of DKK 4,000 for participation in the study. The fee is taxable. The fee covers time consumption, discomfort and travel expenses. The fee is paid by the end of the subject's participation. If the subject is excluded or choose to withdraw from the trial, a fee equal to the part of the study the subject has completed will be paid.
Chr. Hansen A/S is sponsoring the clinical research project with 400.000 DKK and is interested in developing the technology to identify new microbes with probiotic function. The entire amount will be transferred to Gastroenheden, Herlev Hospital and will cover all expenses associated with the clinical study (visit 1, 2 and 3) including e.g. staff salary, use of equipment and consumables.
Adam Baker, Chr. Hansen A/S, Kim B. Jensen, BRIC University of Copenhagen and Albin Sandelin, Department of Biology, University of Copenhagen initiated the project. Support for the down-stream development of the technology has been obtained from the Danish Innovation Foundation (Grand solutions project "NextGenProbio", http://innovationsfonden.dk/da/case/fremtidens-tarmflora-faar-en-hjaelpende-haand), which support the project with 27.588 mill DKK. This will finance salary and consumables for the downstream analysis. Chr. Hansen will also contribute to finance these expenses. Kim Jensen and Albin Sandelin have no financial relationship with Chr. Hansen A/S, nor do any of the research personnel have any relationship with the Innovation Foundation

13. Study Administrative Structure

Chr. Hansen A/S is the Sponsor of the study.

Dr.med Jakob Hendel will organise the operational conduct of the study.

A list of responsible parties involved in this study is enclosed as Appendix 2 to this protocol.

CHR HANSEN
Improving food & health

References

1. Canonici, A., Pellegrino, E., Siret, C., Terciolo, C., Czerucka, D., Bastonero, S., Marvaldi, J., Lombardo, D., Rigot, V., and Andre, F. (2012). Saccharomyces boulardii improves intestinal epithelial cell restitution by inhibiting alphavbeta5 integrin activation state. PLoS One *7*, e45047.

2. Di Giancamillo, A., Vitari, F., Savoini, G., Bontempo, V., Bersani, C., Dell'Orto, V., and Domeneghini, C. (2008). Effects of orally administered probiotic Pediococcus acidilactici on the small and large intestine of weaning piglets. A qualitative and quantitative micro-anatomical study. Histol Histopathol *23*, 651-664.

3. Douillard, F.P., Ribbera, A., Jarvinen, H.M., Kant, R., Pietila, T.E., Randazzo, C., Paulin, L., Laine, P.K., Caggia, C., von Ossowski, I.*, et al.* (2013). Comparative genomic and functional analysis of Lactobacillus casei and Lactobacillus rhamnosus strains marketed as probiotics. Appl Environ Microbiol *79*, 1923-1933.

4. Flint, H.J., Scott, K.P., Louis, P., and Duncan, S.H. (2012). The role of the gut microbiota in nutrition and health. Nat Rev Gastroenterol Hepatol *9*, 577-589.

5. Hsiao, E.Y., McBride, S.W., Hsien, S., Sharon, G., Hyde, E.R., McCue, T., Codelli, J.A., Chow, J., Reisman, S.E., Petrosino, J.F.*, et al.* (2013). Microbiota modulate behavioral and physiological abnormalities associated with neurodevelopmental disorders. Cell *155*, 1451-1463.

6. Jung, P., Sato, T., Merlos-Suarez, A., Barriga, F.M., Iglesias, M., Rossell, D., Auer, H., Gallardo, M., Blasco, M.A., Sancho, E.*, et al.* (2011). Isolation and in vitro expansion of human colonic stem cells. Nature medicine *17*, 1225-1227.

7. Jungersen, M., Wind, A., Johansen, E., Christensen, J.E., Stuer-Lauridsen, B., and Eskesen, D. (2014). The Science behind the Probiotic Strain Bifidobacterium animalis subsp. lactis BB-12. Microorganisms *2*, 92-110.

8. Lebeer, S., Claes, I., Tytgat, H.L., Verhoeven, T.L., Marien, E., von Ossowski, I., Reunanen, J., Palva, A., Vos, W.M., Keersmaecker, S.C.*, et al.* (2012). Functional analysis of Lactobacillus rhamnosus GG pili in relation to adhesion and immunomodulatory interactions with intestinal epithelial cells. Appl Environ Microbiol *78*, 185-193.

9. Li, S.S., Zhu, A., Benes, V., Costea, P.I., Hercog, R., Hildebrand, F., Huerta-Cepas, J., Nieuwdorp, M., Salojarvi, J., Voigt, A.Y.*, et al.* (2016). Durable coexistence of donor and recipient strains after fecal microbiota transplantation. Science *352*, 586-589.

10. Lukovac, S., Belzer, C., Pellis, L., Keijser, B.J., de Vos, W.M., Montijn, R.C., and Roeselers, G. (2014). Differential modulation by Akkermansia muciniphila and Faecalibacterium prausnitzii of host peripheral lipid metabolism and histone acetylation in mouse gut organoids. MBio *5*.

11. Middendorp, S., Schneeberger, K., Wiegerinck, C.L., Mokry, M., Akkerman, R.D., van Wijngaarden, S., Clevers, H., and Nieuwenhuis, E.E. (2014). Adult stem cells in the small intestine are intrinsically programmed with their location-specific function. Stem Cells *32*, 1083-1091.

12. Sakata, T., Kojima, T., Fujieda, M., Miyakozawa, M., Takahashi, M., and Ushida, K. (1999). Probiotic preparations dose-dependently increase net production rates of organic acids and decrease that of ammonia by pig cecal bacteria in batch culture. Dig Dis Sci *44*, 1485-1493.

13. Sato, T., Stange, D.E., Ferrante, M., Vries, R.G., Van Es, J.H., Van den Brink, S., Van Houdt, W.J., Pronk, A., Van Gorp, J., Siersema, P.D.*, et al.* (2011). Long-term expansion of epithelial organoids from human colon, adenoma, adenocarcinoma, and Barrett's epithelium. Gastroenterology *141*, 1762-1772.

14. Ulluwishewa, D., Anderson, R.C., McNabb, W.C., Moughan, P.J., Wells, J.M., and Roy, N.C. (2011). Regulation of tight junction permeability by intestinal bacteria and dietary components. J Nutr *141*, 769-776.

15. van Baarlen, P., Troost, F. J., van Hemert, S., van der Meer, C., de Vos, W. M. de, Groot, P. J., Hooiveld, G. J., Brummer, R. J., Kleerebezem, M. (2011). Differential NF-kappaB pathways induction by Lactobacillus plantarum in the duodenum of healthy humans correlating with immune tolerance. Proc Natl Acad Sci U S A 106, 2371-6.

16. Wang, X., Yamamoto, Y., Wilson, L.H., Zhang, T., Howitt, B.E., Farrow, M.A., Kern, F., Ning, G., Hong, Y., Khor, C.C., *et al.* (2015). Cloning and variation of ground state intestinal stem cells. Nature *522*, 173-178.

17. Yun Chen, Athma A Pai, Jan Herudek, Michal Lubas, Nicola Meola, Aino I Järvelin, Robin Andersson, Vicent Pelechano, Lars M Steinmetz, Torben Heick Jensen & Albin Sandelin (2016). Principles for RNA metabolism and alternative transcription initiation within closely spaced promoters. Nature genetics 48, 984-994

Appendix 1 – Independent Ethics Committee

De Videnskabsetiske Komiteer for Region Hovedstaden
Regionsgården, Kongens Vænge 2, 3400 Hillerød

Telefon: 3866 6395

www.regionh.dk/vek

Appendix 2 – Study Administrative Structure

Lead scientist

Assoc. Prof., PhD Kim B. Jensen

Københavns Universitet, BRIC

Ole Maaløes Vej 5

2200 København N

Clinical Lead:

Dr. med. Jakob Hendel

Gastroenheden, Herlev Hospital

Herlev Ringvej 75

2730 Herlev

Additional participants:

Dr. med. Stine Sloth

Gastroenheden, Herlev Hospital

Herlev Ringvej 75

2730 Herlev

Albin Sandelin

Københavns Universitet, Biologisk Institut

Ole Maaløes Vej 5

2200 København N

Adam Baker

Chr. Hansen A/S

Bøge Alle 10

2970 Hørsholm

Maria Juul Nielsen

Københavns Universitet, BRIC

Ole Maaløes Vej 5

2200 København N

Daniela Kleine-Kohlbrecher

Københavns Universitet, BRIC

Ole Maaløes Vej 5

2200 København N

Christa Broholm

Chr. Hansen A/S

Bøge Alle 10

2970 Hørsholm

Analysis of potential batch effects

MDS analysis on RNA-seq gene expression data from placebo-treated samples showed no substantial dependence on whether the treatments were done on visit 2 or 3 (Supplementary Text 1 Figure 1A) indicating that there was no systematic order of treatments batch effects. There was a slight difference in RNA-seq data between LGG treated samples depending on treatment order (Supplementary Text 1 Figure 1B). This most likely reflects varying degree of responsiveness to LGG (see Results) between the randomly sampled subjects in respective groups rather than a batch effect.

In order to consolidate this hypothesis, we remade subgrouping analysis (Figure 3C) using batch corrected data (limma batch correction of the two visits on top of variance stabilized $\log_2$ fold change between LGG and placebo for every subject and LGG-responding genes). As expected, LGG responses were alleviated due to the batch correction on visits (Supplementary Text 1 Figure 1C), andwe still observed a split between LGG-responsive-and non-responsive subjects, except for five subjects which were in the border zone between these two groups (R5, R8, R10, R12 and R20). To compare with our initial grouping (used in main text), we therefore reassigned the new responder group and non-responder groups using the shrunk clusters (6 subjects out of the original 8 for the new responder group and 12 subjects out of the original 15 for the new non-responder group).

Differential expression analysis was performed within the new responder group and non-responder group. Similar to the results using the original grouping, non-responders had almost no response to LGG (20 up-regulated, 23 down-regulated at FDR<0.05, and only 4 up-regulated genes when also requiring an absolute $\log_2$ fold change >0.5). Responders showed a much larger change:252 upregulated and 550 downregulated genes (*FDR*<0.05), and 108 upregulated and 34 downregulated genes when also requiring an absolute $\log_2$ fold change >0.5) (Supplementary Text 1 Figure 1D, upper bar plots). The majority of the differentially expressed genes found in responders overlapped the ones identified in the original responder group which consisted of 8 subjects (Supplementary Text 1 Figure 1D, lower Venn diagrams).

In agreement with the results of the GO analysis done in the original responder group, genes upregulated after LGG treatment in the new responder group were also strongly enriched for GO terms related to B cell activation, immune system, immune response, and leukocyte activation (Supplementary Text 1 Figure 1E). Similarly, for pathway analysis, KEGG and REACTOME pathway over-representation analysis showed a strong over-representation of pathways related to B cell activation through the B cell receptor (Supplementary Text 1 Figure 1E).

Thus, the results we show in main text are robust even if we model a possible batch effect.

Supplementary Text 1 Figure 1

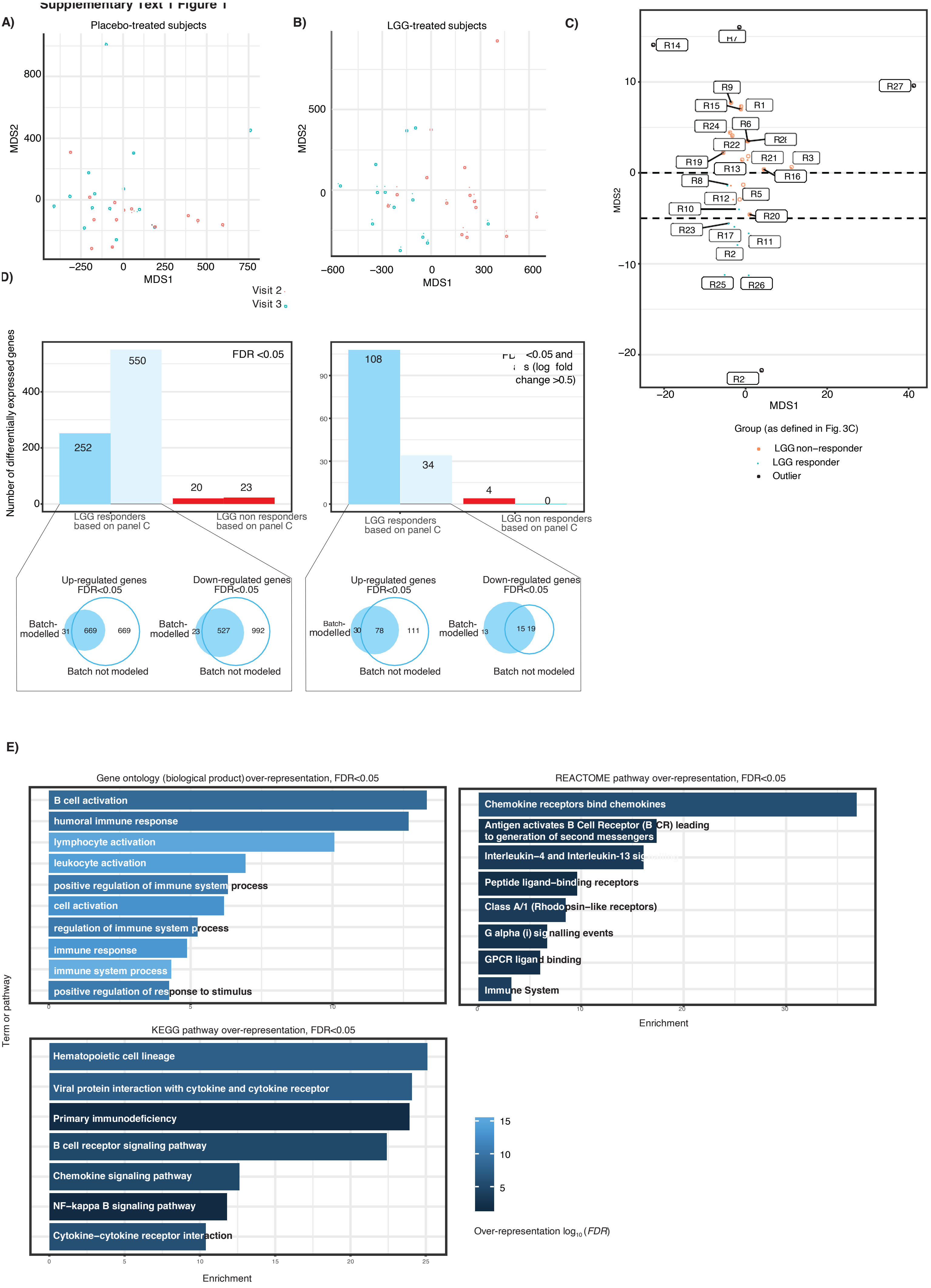

Figure legend for Supplementary Text 1 Figure 1

A: Multidimensional scaling (MDS) analysis of placebo-treated RNA-seq libraries. Axes represent dimensions 1 and 2. Colors indicate which visit the sample was collected from.

B: Multidimensional scaling (MDS) analysis of LGG-treated RNA-seq jejunum libraries. Organized as panel A.

C: Multidimensional scaling (MDS) plot based on log_2 LGG/control RNA-seq expression values. Axes represent dimensions 1 and 2. Dots represent subjects, colored by group: LGG-responders, non-responders and outliers defined in Figure 3C. Labels show subject IDs (see supplementary Data 1) LGG responders and non-LGG responders were observed as separate groups except for five subjects (subject R8 and R10 from LGG-responding group in Figure 3C; R5, R12 and R20 from non-LGG-responding group). These five subjects were therefore excluded from the differential expression analysis in panel D. The subset of genes (*N*=1237) that showed LGG responsiveness were used for MDS analysis, using the same cutoffs as in Figure 3C, as described in Methods).

D: Differential expression analysis and comparison to results from Figure 3. Upper panel shows differential expression analysis using groups defined in panel C. Y axis shows the number of differentially expressed genes (LGG vs placebo) within groups defined in panel C (X axis; group colors as in Figure 3D; opaque colors show up-regulation, pale colors shows down-regulation). Upper left panel shows the results of analysis using $FDR<0.05$ as cutoff, upper right panel shows the results of analysis using $FDR<0.05$ and absolute log_2 fold change >0.5 as cutoffs. Numbers on bars show number of up/down-regulated genes. Lower panel shows Venn diagrams comparing the overlap of differentially expressed genes identified in LGG responders defined using batch modeled data (as above) or non-batch-modeled data (as in Figure 3). Filled circles indicate for batch modeled results and contoured circles strand for non-batch modeled results. Lower left and lower right panels correspond to upper left and upper right panels, respectively.

E: Gene ontology (GO) and pathway analysis of genes upregulated after LGG treatment in the LGG-responder group. The analysis is identical to Figure 4A-C but using up-regulated genes from the batch-corrected data above for over-representation analysis, with the same gene list background.
